# Metal–Halogen Bonding Seen through the Eyes of Vibrational Spectroscopy

**DOI:** 10.3390/ma13010055

**Published:** 2019-12-20

**Authors:** Vytor P. Oliveira, Bruna L. Marcial, Francisco B. C. Machado, Elfi Kraka

**Affiliations:** 1 Departamento de Química, Instituto Tecnológico de Aeronáutica (ITA), São José dos Campos, 12228-900 São Paulo, Brazil; vytor3@gmail.com (V.P.O.); fmachado@ita.br (F.B.C.M.); 2 Núcleo de Química, Instituto Federal Goiano (IF Goiano), Campus Morrinhos, 75650-000 Goiás, Brazil; bruna.marcial@ifgoiano.edu.br; 3 Department of Chemistry, Southern Methodist University, 3215 Daniel Avenue, Dallas, TX 75275-0314, USA

**Keywords:** halogen bond, metal–halogen bond, DFT, CCSD(T), local vibrational modes, local mode force constants and bond strength order

## Abstract

Incorporation of a metal center into halogen-bonded materials can efficiently fine-tune the strength of the halogen bonds and introduce new electronic functionalities. The metal atom can adopt two possible roles: serving as halogen acceptor or polarizing the halogen donor and acceptor groups. We investigated both scenarios for 23 metal–halogen dimers trans-M(Y_2_)(NC_5_H_4_X-3)_2_ with M = Pd(II), Pt(II); Y = F, Cl, Br; X = Cl, Br, I; and NC_5_H_4_X-3 = 3-halopyridine. As a new tool for the quantitative assessment of metal–halogen bonding, we introduced our local vibrational mode analysis, complemented by energy and electron density analyses and electrostatic potential studies at the density functional theory (DFT) and coupled-cluster single, double, and perturbative triple excitations (CCSD(T)) levels of theory. We could for the first time quantify the various attractive contacts and their contribution to the dimer stability and clarify the special role of halogen bonding in these systems. The largest contribution to the stability of the dimers is either due to halogen bonding or nonspecific interactions. Hydrogen bonding plays only a secondary role. The metal can only act as halogen acceptor when the monomer adopts a (quasi-)planar geometry. The best strategy to accomplish this is to substitute the halo-pyridine ring with a halo-diazole ring, which considerably strengthens halogen bonding. Our findings based on the local mode analysis provide a solid platform for fine-tuning of existing and for design of new metal–halogen-bonded materials.

## 1. Introduction

Halogen bonding (XB) is a non-covalent interaction formed between the electrophilic region of a halogen atom (X) in a halogenated moiety, called halogen donor (DX), and the nucleophilic region of a partner molecule (moiety) called halogen acceptor (AR${}_{n}$), where A is usually a heteroatom with substituents R${}_{n}$ [1,2]. XB was first experimentally observed in 1863 by Guthrie for I_2_…NH_3_ complexes [3]. In the 1950s, the existence and characterization of XB was established by Mulliken’s groundbreaking theoretical work on charge-transfer interactions [4,5,6] and was supported by crystallographic studies of Hassel and coworkers [7]. However, it took the scientific community until the 1980s to fully recognize the importance of XB across diverse fields of chemistry and biology [8,9,10], including chemical engineering and materials science [11,12], in particular discovering the potential of XB in guiding self-assembly [13,14], which goes far beyond the formation of hydrogen bond (HB) analogs.

Halogen bonds (also abbreviated with XB in the following) share several common features with HBs [15,16,17,18]. Similar to the HB, the XB tends to adopt a linear arrangement between DX and A (denoted by D-X…A) [19,20,21]. However, in contrast to a hydrogen atom, a monovalent halogen is amphoteric: (i) collinear with but on the opposite side of the σ-bond, the halogen atom acts as a Lewis acid due to the formation of an electron-depletion region, σ-hole region [22,23,24] in which the nucleus is less shielded; (ii) orthogonal to the σ-bond, i.e., in the π-region, electron density is accumulated due to the halogen lone pairs, allowing the halogen to act as a Lewis base, i.e., as a halogen or hydrogen acceptor.

A halogen bond strong enough to effectively guide self-assembly [13] requires a polarizable X atom (usually bromine or iodine) paired to a σ-electron-withdrawing group at DX, which strongly polarizes the XB by increasing the positive electrostatic potential at the σ-hole [22,23,24]. For this purpose, halogens are often paired with alkynes, fluorinated alkenes, alkanes and benzenes, or halopyridines [8], in which the nitrogen magnifies the electron-withdrawing effect of the ring [25]. Further polarization of the halogen in halopyridines can be achieved by protonation, by methylation [26], or by coordinating the nitrogen atom to a metal center [26,27]. The strength of the XB also depends on the electron donor ability of the halogen-acceptor A, which can be a heteroatom, an anion, or the π-density of an unsaturated system [16]. The acceptor capability of a halogen to engage in HB or an XB can be enhanced by having it bonded to a metal instead of a carbon due to the greater polarity of the M–X bond compared to the C–X bond [25,27] as already suggested by Koten and coworkers in 1986 [28].

Noticing that a metal center can improve both the donor ability of the DX moiety and the halogen acceptor ability of a halogen directly bonded to the metal, Zordan and coworkers [25] systematically studied the crystal structure of trans-M(Cl_2_)(NC_5_H_4_X-3)_2_ with M = Pd(II) and Pt(II); X = F, Cl, Br, I; and NC_5_H_4_X-3 = 3-halopyridine in order to establish the applicability of the M–Cl…X–C halogen bonds to drive supramolecular self-assembly. They found that a network of XBs propagate in one-dimensional tape-like structures or two-dimensional layer structures depending on the metal, the halogen, and the synthetic strategy. Noteworthy is that no XBs were observed for X = F. This pioneering work has served as the basis for the design of a large number of metal-containing XB materials with a halopyridine derivative attached to the halogenated metal center [29,30,31,32,33,34,35,36,37].

Supramolecular self-assembly and crystallization processes are a result of a complex interplay of many non-covalent interactions [38]. Although difficult to predict, these processes can be facilitated by using molecular systems capable of forming strong and directional non-covalent interactions such as halogen and hydrogen bonding [8]. Hence, there has been an increasing effort to classify the strength of non-covalent interactions in small systems that could be used as building blocks for self-assembly of supramolecular structures on the basis of theoretical models and computational tools [16,39]. Popular computational approaches utilized to determine the strength of a chemical bond or weak chemical interaction include molecular orbital approaches [40,41] or energy decomposition methods [42,43,44]. However, these approaches also provide more qualitative rather than quantitative results [45,46]. Other approaches are based on the inspection of the electrostatic potential [47], the topological analysis of the electron density via Bader’s quantum theory of atoms in molecules (QTAIM) approach [48,49], or on the inspection of a few geometric parameters [50]. Although molecular electrostatic potentials can serve as a suitable tool to predict the crystal arrangement based on simple electrostatic arguments, they face limitations when used to predict the strength of an interaction or chemical bond [16]. The topological analysis of the electron density can be useful to uncover possible attractive contacts between two atoms via the existence of a maximum electron density path (called bond path) with a bond critical point (BCP) connecting the two nuclei under consideration [48,51]. However, the sole existence of a bond path and a BCP does not necessarily imply the existence of a chemical bond [52]. For distant atoms, bond paths may favor a connection between electron-rich regions instead of reflecting dominant interactions, making the interpretation of bond paths as bond indicators and the use of the density at the BCP as bond strength measure problematic [52,53,54]. The same holds for bond strength measures derived from dissociation energies (BDE)s [55,56,57,58,59,60] and molecular geometries. While these approaches have certainly contributed to the chemical understanding of chemical bondings, one has to realize that BDE values or bond lengths provide little insight into the intrinsic strength of the bond. The BDE is a cumulative quantity recording the overall energy required for the dissociation of a molecule into fragments. Accordingly, it includes any (de)stabilization effects of the products to be formed. The magnitude of the BDE reflects the energy needed for bond breaking and contains energy contributions due to geometry relaxation and electron density reorganization in the dissociation fragments. Therefore, the BDE is not a suitable measure of the intrinsic strength of a chemical bond as it is strongly affected in non-predictable ways by the changes of the dissociation fragments. Accordingly, its use has led in many cases to a misjudgment of bond strength [16,45,61,62,63,64]. Also, the bond length is not a qualified bond strength descriptor. Numerous cases have been reported illustrating that a shorter bond is not always a stronger bond [65,66,67,68,69].

On the other hand, detailed information on the electronic structure of a molecule and its chemical bonds is encoded in the molecular normal vibrational modes [70]. Therefore, vibrational spectroscopy data can serve as a powerful source for exploring what are the main contributions to bonding and weak chemical interactions in a compound, which can be due to specific atom–atom interactions such as XB and HB [9] or less specific interactions such as π–π-stacking [71] or halogen–π interactions [72]. Detailed knowledge of these contributions from the prerequisites for achieving greater control over supramolecular architectures and for the fine-tuning and design of new materials is needed. Therefore, we introduce in this work a new quantitative measure for the intrinsic bond strength of metal–halogen bonding based on the local vibrational mode analysis first introduced by Konkoli and Cremer [67,68,73,74]. The investigation of 23 metal–halogen complexes derived from the dimers of Zordan and coworkers [25] focused in particular on answering the following questions:

1. Is XB the most attractive interaction present in these systems? How strong is it compared to other non-covalent interactions present such as HB and π-stacking between pyridine rings or other interactions being overlooked so far?
2. What is the best strategy to fine-tune the strength of the XB in these systems: changing the halogen, the halogen-donor group DX, or the metal or halogen of the halogen-acceptor A?

## 2. Computational Methods and Procedures

In the following calculational details, the software used and the theory of the local mode analysis will be described.

### 2.1. Calculational Details

**Figure 1:**
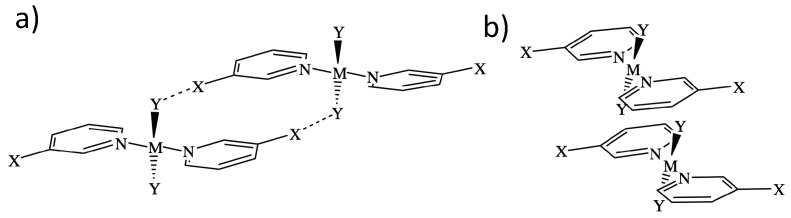
Representation of the two different binding modes studies: Dimer 1 is held by halogen bonds between X and Y (**a**), and dimer two is held by π-stacking interactions (**b**). M is a metal (Pt or Pd); X and Y are halogen atoms.

**Figure 2:**
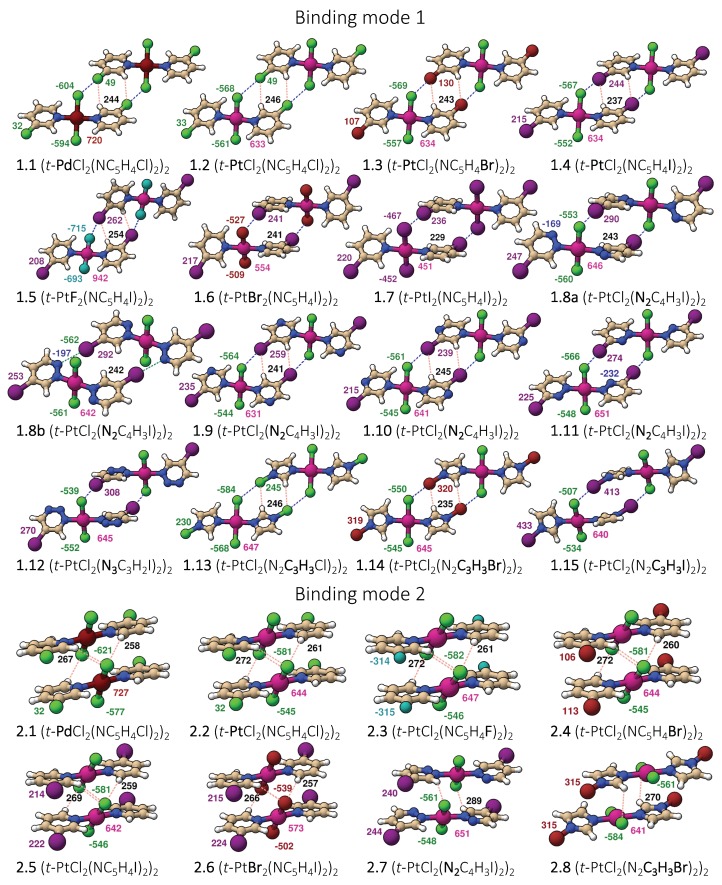
Representation of all dimers studied with color coded atomic charges from natural population analysis (NPA): atom–atom interactions are given by dashed lines.

Two representative dimer binding modes were investigated, derived from crystal structures reported by Zordan et al. [25]. Binding mode 1 is formed by a dimer held together by two halogen bonds, whereas binding mode 2 is a π-stacking dimer (see Figure 1). Both binding modes are of $C_{i}$ symmetry, and beside halogen and π-stacking, a few short HB contacts are also present [25]. To find the best strategy for fine-tuning the strength of the halogen bonds in binding mode 1, we systematically modified the monomer trans-M(Y_2_)(NC_5_H_4_X-3)${}_{2}$ by varying the metal M = Pd and Pt, the halogen at the halo-pyridines (X = Cl, Br, and I), the halogens at the metal (Y = F, Cl, Br, and I), and the pyridine ring, resulting in 15 different structures numbered from **1.1** to **1.15**. Several of these modification were also tested for binding mode 2, generating 8 additional structures numbered from **2.1** to **2.8** leading to a total of 23 dimers shown in Figure 2. To clarify the different roles the metal may adopt, we started from three monomers, positioned an I_2_ molecule close to the metal at different orientations, searched for possible XBs with the d${}_{z^{2}}$ lone pair orbital of Pt(II) and Pd(II), commonly observed in similar systems [35,75,76,77,78,79,80] or interactions, with the π density of the I_2_ leading to six different structures.

All geometries optimizations and subsequent harmonic vibrational frequency calculations were performed with the Chai and Head–Gordon long-range corrected hybrid functional ωB97XD [81] with Grimme’s empirical dispersion correction [82]. This functional is known to reliably describe non-covalent interactions [83]. As a basis, set we used a modified version of Dunning’s augmented valence triple-ζ basis set, termed jun-cc-pVTZ [84,85], where H diffuse functions and the highest angular momentum diffuse functions are removed to decrease computational costs. For Br and heavier elements, we used relativistic effective core potentials (RECP) matched with aug-cc-pVTZ-PP basis sets [86,87,88,89]. Tight convergence criteria were utilized for the self-consistent field equations and geometry optimizations combined with a superfine integration grid to guarantee an accurate description of the weak interactions [90].

Binding energies (ΔE), calculated as the sum of the interaction energy and geometry relaxation energies of the monomers were computed at two different levels of theory: (i) at the ωB97XD level with and without counterpoise correction (CP) [91,92] for the interaction energy and (ii) at the coupled-cluster (CCSD(T)) level of theory [93] utilizing the domain-based local pair natural orbital (DLPNO) approximation [94], DLPNO-CCSD(T), combined with the augmented triple-ζ basis set ma-def2-TZVP [95,96]. The DLPNO approach uses localized PNOs and projection techniques to exploit the locality of electron correlation. Computational costs are reduced by truncating the virtual space and by neglecting the correlation between distant pairs or by treating this correlation at a lower level of theory depending on the orbital overlap. For an optimal balance between accuracy and computational costs, normal PNO accuracy settings [97] and def2-TZVPD/C auxiliary basis sets [98] were utilized. The DLPNO approximation is able to provide near CCSD(T) energies (estimated mean absolute deviation from canonical CCSD(T) ΔE values are of 0.3 kcal/mol) at a small fraction of the computational costs [97,99]. Possible attractive contacts were determined with the topological analysis of the electron density [48]. In order to quantify the covalent/electrostatic character of these interactions, we applied the Cremer–Kraka criterion of covalent bonding [16,100,101,102]. According to this criterion, a covalent bond between two atoms A and B is defined by the following two conditions; (i) *Necessary condition*: The existence of a bond path and BCP $r_{b}$ = *b* between A and B; (ii) *sufficient condition*: The energy density $H(r_{b})$ = $H_{b}$ is smaller than zero. H(r) is defined as follows:

(1) H(r)=G(r)+V(r)

where G(r) is the kinetic energy density and V(r) is the potential energy density. The negative V(r) corresponds to a stabilizing accumulation of density whereas the positive G(r) corresponds to depletion of electron density [101]. As a result, the sign of $H_{b}$ indicates which term is dominant [103]. If $H_{b}<0$, the interaction is considered covalent in nature, whereas $H_{b}>0$ is indicative of electrostatic interactions.

Considering the problem of finding bond paths connecting electron-rich regions instead of reflecting dominant interactions [53,54], the topological electron density analysis was complemented by electron difference densities. To distinguish an atom–atom interaction from a dispersive/electrostatic interaction involving multiple atoms, termed here nonspecific (NS), we calculated and analyzed reduced density gradient (RDG) iso-surfaces of 0.45 a.u., colored according to the value of the second largest eigenvalue of the electron density Hessian, also termed non-covalent index (NCI) [104].

As a new quantitative measure of the intrinsic strength of the metal–halogen interactions and their comparison with other weak interactions characterizing the compounds investigated in this work, local mode force constants derived from local vibrational modes were used [45,46,67,68,73,74]. In the following, the underlying theory will be described.

### 2.2. The Theory of Local Vibrational Modes

ΔE values account for all possible attractive and repulsive contacts present and, therefore, cannot be used to compare the strength of individual atom–atom contributions. On the other hand, detailed information on the electronic structure of a molecule and its chemical bonds is encoded in the normal vibrational modes. Therefore, vibrational spectroscopy could serve as an excellent source for an intrinsic bond strength measure via the bond-stretching frequency $\omega_{\mu }$ and in particular via the associated force constant k${}_{\mu }$.

The idea of characterizing the bond strength via the stretching force constant began in the 1920s and 1930s with the famous Badger’s rule [105], which states that the strength of a bond correlates with the frequency and related force constant of its vibrational mode. Over the next decades, this rule was frequently applied, stretching from the early work of Gordy on the relationship between bond force constants, bond orders, bond lengths, and electronegativity of the bond partners [106] to the work of Legon on the discussion of bond force constants for halogen complexes [107]. A comprehensive, historic overview can be found in a review article by Kraka, Larsson, and Cremer [108]. However, many applications revealed that, while Badger’s original rule works fine for diatomic molecules, its extension to polyatomic molecules is problematic; the vibrational modes of polyatomic molecules are generally delocalized because of mode–mode coupling and, therefore, do not lead to stretching force constants which may serve directly as bond strength measure [70,108,109].

There are two different coupling mechanisms between vibrational modes, i.e., *mass coupling* reflected by the kinetic and *electronic coupling* reflecting the potential energy contribution to the molecular vibration [70,110], which can be conveniently described by Lagrangian mechanics.

(2)L(x,x˙)=T(x˙)−V(x)(3)=12x˙†Mx˙−12x†Fxx

where $L(x,\dot{x})$ is the Lagrangian defined as the difference of the kinetic energy $T(\dot{x})$ and the potential energy V(x). x is a vector with 3N Cartesian displacement coordinates of a molecule being composed of N atoms. $\dot{x}$ contains the Cartesian velocity elements $\frac{\delta x_{i}}{\delta t}$. $F^{x}$ is the force constant matrix (Hessian) in Cartesian coordinates x, with **M** as the mass matrix, a diagonal matrix containing each atomic mass three times to account for motion in the x, y, and z directions. Both square matrices have the dimension (3N×3N). The Cartesian displacement coordinates describe the deviation from the equilibrium position $x_{e}$, with

(4) $$\begin{array}{ccc} \Delta x & = & x-x_{e}\equiv x \end{array}$$

The potential energy V(x) is zero when the atoms are in their equilibrium position and greater than zero otherwise.

A solution to Equation (2) can be obtained via the Euler–Lagrange equations:

(5) $$\begin{array}{c} \frac{L(x,\dot{x})}{\partial \dot{x_{i}}}-\frac{\partial L(x,\dot{x})}{\partial x_{i}}=0\;\;\;i=1,\ldots ,3N \end{array}$$

The Lagrangian can be also expressed in terms of $N_{vib}=(3N-\Sigma$) internal coordinates q; (Σ: number of translations and rotations; 6 for nonlinear and 5 for linear molecules). This leads to the following Lagrangian:

(6)L(q,q˙)=T(q˙)−V(q)(7)=12q˙†G−1q˙−12q†Fqq

where $F^{q}$ is the force constant matrix in internal coordinates q and **G** is the Wilson **G** matrix, [70] also called “inverse kinetic energy” matrix. Both square matrices have the dimension $\left(3N_{vib}\times 3N_{vib}\right)$.

The relationship between internal and Cartesian coordinates is provided by the Wilson B matrix, a rectangular ($N_{vib}\times 3N$) matrix containing the first derivatives of the internal coordinates $q_{n}(n=1,2,3\ldots N_{vib}$) with regard to the Cartesian coordinates $x_{i}(i=1,2,3\ldots 3N$):

(8) $$\begin{array}{ccc} q & = & Bx \end{array}$$

(9) $$\begin{array}{ccc} B_{n} & = & \frac{\delta q_{n}\left(x\right)}{\delta x_{i}} \end{array}$$

The transformation between internal and Cartesian coordinates via Equations (8) and (9) can be extended to puckering coordinates, symmetry coordinates, or other special coordinates, e.g., coordinates describing dummy atoms as long as Equation (9) is defined for the coordinates under consideration.

The electronic coupling of the vibrational modes can be suppressed by solving the Wilson equation of vibrational spectroscopy and, in this way, by transforming to normal coordinates **Q** [70,111,112]. In Equation (10), the Wilson equation of vibrational spectroscopy is given [70,111,112]:

(10) $$\begin{array}{c} F^{x}L=ML\Lambda \end{array}$$

where matrix L collects the vibrational eigenvectors $l_{\mu }$ in its columns. Λ is a diagonal matrix with the eigenvalues $\lambda_{\mu }$, which leads to the (harmonic) vibrational frequencies $\omega_{\mu }$ according to $\lambda_{\mu }=4\pi^{2}c^{2}\omega_{\mu }^{2}$. The number of vibrational modes is given by $N_{vib}$, i.e., translational and rotational motions of the molecule are already eliminated. Diagonalization of force constant matrix $F^{x}$ according to $L^{†}F^{x}L=\Lambda$ leads to the diagonal normal force constant matrix $F^{Q}$ = K given in normal coordinates Q, i.e., a force constant matrix without electronic coupling.

Expressing the molecular geometry in terms of internal coordinates $q_{n}$ rather than Cartesian coordinates $x_{n}$, the Wilson equation adopts a new form [70]:

(11) $$\begin{array}{c} F^{q}D=G^{-1}D\Lambda \end{array}$$

where D collects the normal mode vectors $d_{\mu }$ ($\mu =1,\ldots ,N_{vib}$) column-wise and matrix $G=BM^{-1}B^{†}$ (Wilson G-matrix) gives the kinetic energy in terms of internal coordinates [70]. The eigenvector matrix D has the property to diagonalize $F^{q}$, leading to the diagonal force constant matrix $D^{†}F^{q}D=K$.

After the elimination of the electronic coupling of the vibrational modes, there is still the mass- coupling represented by the off-diagonal G matrix elements, which often has been overlooked [45,46]. Konkoli and Cremer derived local vibrational modes which are free of both electronic and mass coupling directly from normal vibrational modes by solving mass-decoupled Euler–Lagrange equations [67,68,73,74]. Each local mode is associated with an internal coordinate $q_{n}$, which drives the local mode, i.e., only the masses of the atoms fragment $\phi_{n}$ involved in the local vibration being described by $q_{n}$ are nonzero and the masses of all other *m* not involved atoms are zero so that they can effortlessly follow the vibration led by $q_{n}$. In this way, Equation (5) adapts the following form:

(12) $$\begin{array}{c} \begin{array}{cc} p_{n}=\frac{\delta L(q,\dot{q})}{\delta {\dot{q}}_{n}}\neq 0, & \frac{d}{dt}p_{n}=\frac{\delta V(q)}{\delta q_{n}}\neq 0\\ p_{m}=\frac{\delta L(q,\dot{q})}{\delta {\dot{q}}_{m}}=0, & \frac{d}{dt}p_{m}=\frac{\delta V(q)}{\delta q_{m}}=0 \end{array} \end{array}$$

The solution of Equation (12) leads to the local mode vector $a_{n}$ associated with the internal coordinate $q_{n}$ describing the *n*th local mode [67,68,73,74]:

(13) $$a_{n}=\frac{K^{-1}d_{n}^{†}}{d_{n}K^{-1}d_{n}^{†}}$$

i.e., what is needed for the local mode analysis is the diagonal force constant K in normal coordinates **Q** and the row vectors **d**${}_{n}$ of the matrix D. The associated local mode force constant $k_{n}^{a}$ of mode *n* (superscript *a* denotes an adiabatically relaxed, i.e., local mode) is obtained via Equation (14):

(14) $$k_{n}^{a}=a_{n}^{†}Ka_{n}={\left(d_{n}K^{-1}d_{n}^{†}\right)}^{-1}$$

Local mode force constants, contrary to normal mode force constants, have the advantage of being independent of the choice of the coordinates used to describe the molecule in question [66,73]. In recent work, Zou and coworkers proved that the compliance constants $\Gamma_{nn}$ of Decius [113] are simply the reciprocal of the local mode force constants: $k_{n}^{a}=1/\Gamma_{nn}$ [46,114].

In order to facilitate the bond strength discussion for a larger series of molecules, it is convenient to refer to a relative bond strength order (BSO) *n* rather than to directly compare local force constant values. Both are connected via a power relationship according to the generalized Badger rule derived by Kraka and coworkers [108] They could show that, with this extended Badger rule, different bonds between atoms of the same period can be described by one common power relationship of the following form:

(15) $$BSO\;n=a\;\left(k^{a}\right){\;}^{b}$$

The constants *a* and *b* in Equation (15) can be determined via two reference compounds with known $k^{a}$ values and the requirement that, for a zero force constant, the BSO *n* is zero. In this work, we used as reference molecules I_2_, two center-two electron bond (2c-2e) with $k^{a}$ 1.936 (mDyn/Å) and BSO *n* 1.0, and I_3_, three center-four electron bond (3c-4e) with $k^{a}$ 0.556 (mDyn/Å) and BSO *n* 0.5.

The reduced mass of the local mode $a_{n}$ is given by the diagonal element $G_{nn}$ of the **G**-matrix [73]. Local mode force constant and mass are needed to determine the local mode frequency $\omega_{n}^{a}$:

(16) $${\left(\omega_{n}^{a}\right)}^{2}=\frac{1}{4\pi^{2}c^{2}}k_{n}^{a}G_{nn}$$

Apart from these properties, it is straightforward to determine the local mode infrared intensity or the Raman intensity [115].

The local mode analysis has been successfully applied to characterize covalent bonds [63,65,108,116,117,118,119] and weak chemical interactions such as halogen [15,16,80,120], chalcogen [17,102,121], pnicogen [122,123,124], and tetrel interactions [64] as well as H-bonding [18,125,126,127,128,129] and BH…π interactions [130,131]. A new metal-ligand electronic parameter (MLEP) was derived as quantitative measure of the intrinsic strength of metal–ligand bonding [129,132,133,134,135]. Recently, the local mode analysis was for the first time successfully applied to periodic systems [136].

### 2.3. Software Used

All density functional calculations were carried out with program package Gaussian 16 [137]. DLPNO-CCSD(T) single-point energy calculations were performed with Orca 4.1 [138,139]; the electron density analysis, electrostatic potentials, electron difference densities, and NCI plots were computed with Multiwfn [140]. The local mode analysis was performed with Cologne2019 [141]. For the assessment of nonspecific interactions (NS) between an atom and a bond, the local stretching mode and associated force constant were computed between the atom and the bond midpoint; for interactions involving an atom and aromatic ring, the local stretching mode was calculated between the atom and the normal to the ring plane. In both cases, the corresponding B matrix elements were coded.

## 3. Results and Discussion

**Figure 3:**
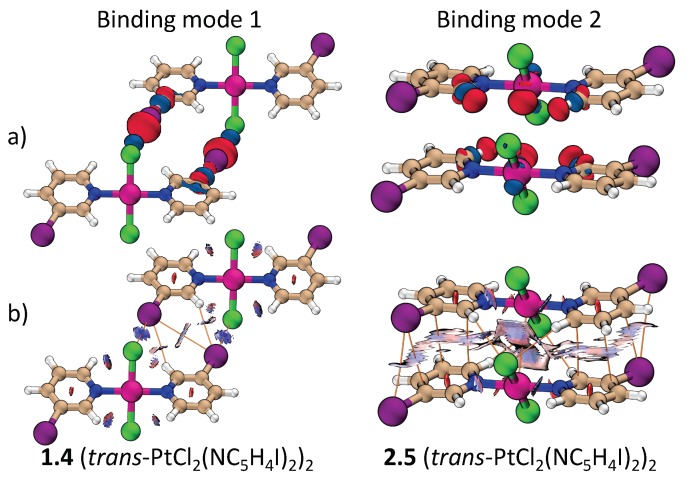
Comparison of binding mode 1 and 2 for the (t-PtCl_2_(NC_5_H_4_I)_2_)_2_ dimer: (**a**) The electron difference densities and (**b**) the colored reduced density gradient (RDG) and bond paths.

Figure 2 shows the dimers studied in this work. They are separated into two groups according to their binding mode as defined in Figure 1. Attractive interactions present in each binding mode are highlighted in Figure 2 by dashed lines. Figure 3 contains the positive and negative 0.001 a.u. electron difference (iso)densities (EDD) of (trans-PtCl_2_(NC_4_H_4_I)_2_)_2_ (Figure 3a), left-side binding mode 1, and right-side binding mode 2. Red regions indicate electron depletion upon complexation, whereas blue regions indicate electron accumulation. Figure 3b show both the corresponding bond paths (orange lines) and the 0.45 RDG isodensity colored according to the values of the second largest eigenvalue of the electron density Hessian ($\lambda_{2}$). Dark blue regions ($\lambda_{2}<$ 0) indicate attractive interactions, light color ($\lambda_{2}\approx$ 0) indicates a dispersive interaction, and red region ($\lambda_{2}>$ 0) indicates steric repulsion.

According to Figure 3, the stability of binding mode 1 can be attribute to a C–X…X’-Pt XB and a C-H…X–C HB. Unexpectedly, a bond path connecting C–X…X–C is also observed even though the distance between the halogens are greater than the sum of their van der Waals radii. There may be a very weak dispersive interaction between these halogens, but the X…X bond path may also be a consequence of the increased electron density at the π region of the halogens stabilized by the C-H…X–C HB [53]. Either way, this interaction plays little to no role for the stabilization of the dimers.

In binding mode 2, the dimers are arranged in a way that maximizes electrostatic/dispersive attraction and minimizes Pauli/electrostatic repulsion. The halo-pyridine rings are almost parallel but not centrosymetrically arranged. The translation between parallel halo-pyrine rings increases with the size of the halogen substituent. For X = Br and I, the interaction is largely between the C–X bond and the halo-pyridine ring (termed (C–X)…(C_5_N)). Besides halo-pyridine stacking, the bond paths and difference densities in Figure 3 indicate the presence of weak HBs between the halo-pyridines and the Cl atom at the metal. A bond path connecting the two metal atoms is also present in binding mode 2. Since M…M and M…X distances are alway larger than the M…(X–M) distance, where (X–M) indicates the midpoint of the X–M bond, electrostatic/dispersive interaction between M and XM is more likely to take place than a M…M or M…X interaction. This is also confirmed by the light blue region in the RDG and a larger k${}^{a}$ value associated to M…(X–M) compared to M…M or M…X. The formation of an M…M bond path could be a result of the accumulation of density at the d${}_{z^{2}}$ lone pairs, as evidenced by the EDD (Figure 3).

### 3.1. Bond Strength and Binding Energies

Properties related to the most relevant attractive interactions for dimers **1.1**–**1.15** are given in Table 1. Similar information for dimers **2.1**–**2.8** is provided in Table S1 of the Supplementary Materials. Table 1 contains interatomic distances and sum of van der Waals radii taken from the Bondi scale [142] in Å, k${}^{a}$ values for XBs and HBs in mdyn/Å, electron density $\rho_{b}$ at the BCP in e/Å${}^{3}$, and the energy density $H_{b}$ at the same point in Hartree/Å${}^{3}$.

**Figure 4:**
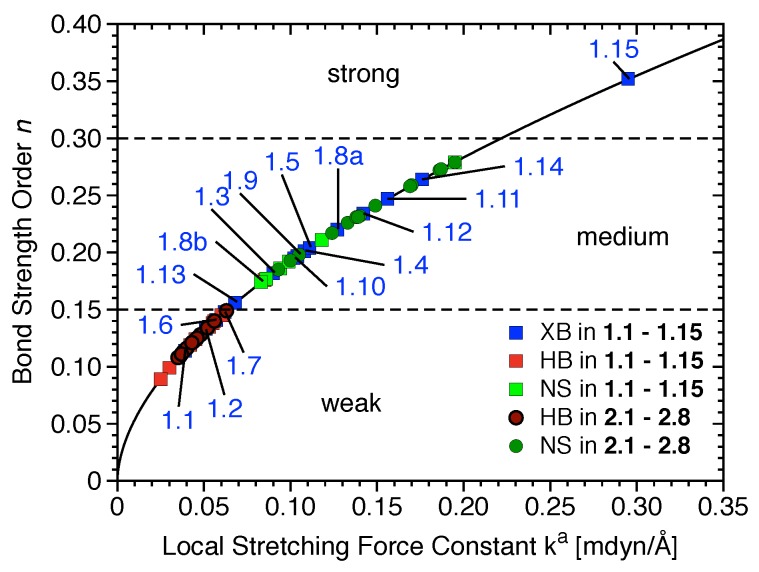
Power relationship between the relative BSO *n* and k${}^{a}$ values for halogen bonds (XBs) of **1.1**–**1.15** (blue), hydrogen bonds (HBs) of **1.1**–**1.15** (red) and **2.1**–**2.8** (dark red), and nonspecific contacts (NS) of **1.1**–**1.15** (green) and **2.1**–**2.8** (dark green).

The most relevant attractive interactions present in **1.1**–**2.8** are ordered according to their strength in the diagram presented in Figure 4. All HBs and the XBs in **1.1**, **1.2**, **1.6**, and **1.7** are located in the lower part of Figure 4, i.e., these are weak interactions (BSO *n*< 0.15), π-stacking, and other nonspecific interactions; most halogen bonds are of intermediate strength (0.15 < BSO *n* < 0.30), and only the halogen bond in **1.15** is strong (BSO *n* > 0.30).

**Figure 5:**
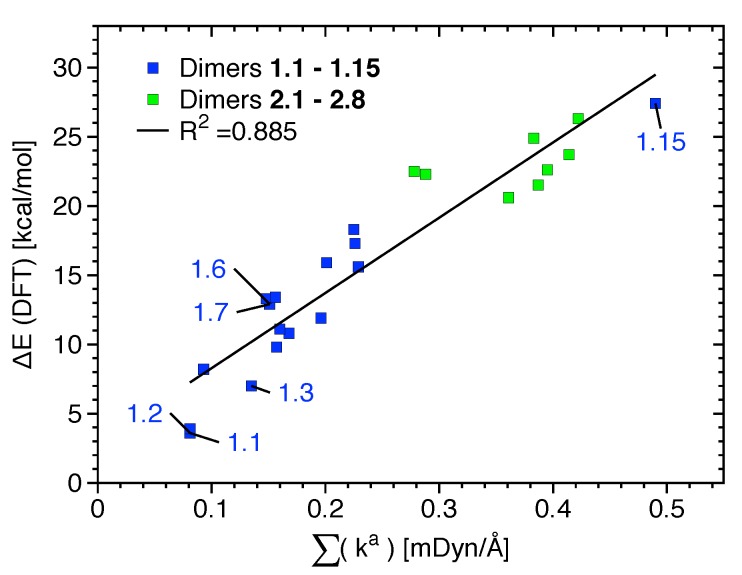
Linear relationship between the sum of k${}^{a}$ values referring to the most significative interactions present in each dimer of binding mode 1 (blue) and 2 (green).

Binding energies computed at the ωB97XD and DLPNO-CCSD(T) levels of theory are provided in Table 2. They give qualitatively similar results (see the bar diagrams of Figures S1 and S2 of the Supplementary Materials); the only noticeable exceptions are that ωB97XD overestimates the ΔE of dimer **1.14** and underestimates the ΔE of **2.1**. No relationship between the strength of the XB given by k${}^{a}$ or BSO *n* values and ΔE values (given in Table 2) are observed for **1.1**–**1.15**, indicating that other non-covalent interactions such as HB and NS are accumulated in ΔE (see also Figure S3 of the Supplementary Materials). If the most relevant non-covalent interactions given in Table 1 and Figure S3 are considered to be approximately independent, i.e., neglecting possible cooperative and anti-cooperative contributions, a relationship between the sum k${}^{a}$ and ΔE could be expected. Indeed, there exists a linear but scattered relationship between ΔE and the sum of k${}^{a}$, as shown in Figure 5. The scattering is not only due to the independent behavior assumed but also due to less relevant attractive/repulsive contacts not being accounted for and due to small changes in the monomer geometry and electron density that takes place upon dimer formation. This is in line with previous studies showing that, because of its accumulative nature, ΔE is not suited as a bond strength measure [45]. If, instead of ΔE, interaction energies (energy difference between the dimer and its unrelaxed monomers) are correlated with the sum of k${}^{a}$, the R${}^{2}$ value increases from 0.885 to 0.906. Although for most monomers the geometry deformation energy is about 1 kcal/mol or less, it reaches a value of 4.6 kcal/mol for the strongest complex (**1.15**).

### 3.2. Strengthening XB in Binding Mode 1

Changing the metal from Palladium **1.1** to Platinum **1.2** revealed little impact on the XB strength, the latter being slightly shorter (by 0.02 Å) and stronger (BSO *n* increases by 0.019), even though the NPA values indicates a higher concentration of electron density on the Cl donor of **1.1**. As expected, a larger effect on binding energy and XB strength results in a more polarizable halogen in the halo-pyridine. This is due to the increase in magnitude of the positive electrostatic potential in the σ-hole region [22]. In line with this increase, X = Cl (**1.2**) < Br (**1.3**) < I (**1.4**), ΔE and BSO *n* values increase, i.e., ΔE = 5.0 (**1.2**), 6.7 (**1.3**), or 10.1 kcal/mol (**1.4**) and BSO *n* = 0.133 (**1.2**), 0.182 (**1.3**), or 0.201 (**1.4**). If Y = Cl is replaced by F, the most electronegative halogen, the negative charge at the halogen acceptor decreases from −567 me to −715 me but the halogen bond strength barely increases, BSO *n* = 0.204 (**1.5**). Due to the smaller size of F, monomer **1.5** adopts a planar geometry, making the halogen acceptor less accessible for the halogen donor. Heavier halogens (X = Br in **1.6** and I **1.7**) have higher NPA charges (−527 me and −467 me), i.e., they are weaker halogen acceptors. Unexpectedly, the binding energy of these complexes, with ΔE = 13.7 (**1.6**) and 13.5 kcal/mol (**1.7**), respectively, is 3.6 and 3.4 kcal/mol larger than that found for **1.4**, which can only be attribute to stronger nonspecific interactions (see Figure S4). An inspection of the geometries of **1.6** and **1.7** showed that the halo-pyridines of each monomer rotate, facilitating the electrostatic interaction between the negative charge in the π-region of the iodine and the positive charge at the C–H bond of the nearby halo-pyridine (see Figure 2). The energetic cost of rotating one halo-pyridine ring about 50 degrees decreases from Y = Cl to I and is of the order of 1 kcal/mol for **1.6** (shown in Figure S5).

Further increase in XB can be achieved by increasing the polarizing power of the iodo-pyridine. Thus, we substituted a CH unit of the iodo-pyridine with a N atom at various ring positions, leading to dimers **1.8**–**1.11**. Binding energies and XB strengths follow a similar order for this series (ΔE(CCSD(T)) = 17.2 (**1.8a**), 13.5 (**1.11**), 10.5 (**1.9**), and 9.7 kcal/mol (**1.10**) and BSO *n* = 0.247 (**1.11**), 0.220 (**1.8a**), 0.197 (**1.09**), and 0.195 (**1.10**) except for **1.11**, which has the strongest XB but lacks a HB present in the other complexes. This shows that, although XB is the major stabilizing factor, secondary contributions from weak HBs and nonspecific contacts are important. Complex **1.8** has two different minima, one involving an XB between the iodo-pyridine and the chlorine atom at the metal (**1.8a**; BSO *n* = 0.220) and another one involving an XB between the iodo-pyridine and the nitrogen added to the halo-pyridine (**1.8b**; BSO *n* = 0.174). The latter has a weaker XB due to the higher charge of the N compared to the Cl (NPA: −197 (N) and −553 (Cl) me). However, the existence of a bond path connecting I to Cl suggests that dispersive interactions may be responsible for the similar binding energies of **1.8a** and **1.8b** (Table 1 and Figure S4). Substitution of a subsequent CH unit in the halo-pyridine ring with N led only to a marginal increase in the XB BSO *n* (**1.12**; BSO *n* 0.234) and in the ΔE (**1.12**; ΔE = 18.0 kcal/mol), compared to **1.8a**.

**Figure 6:**
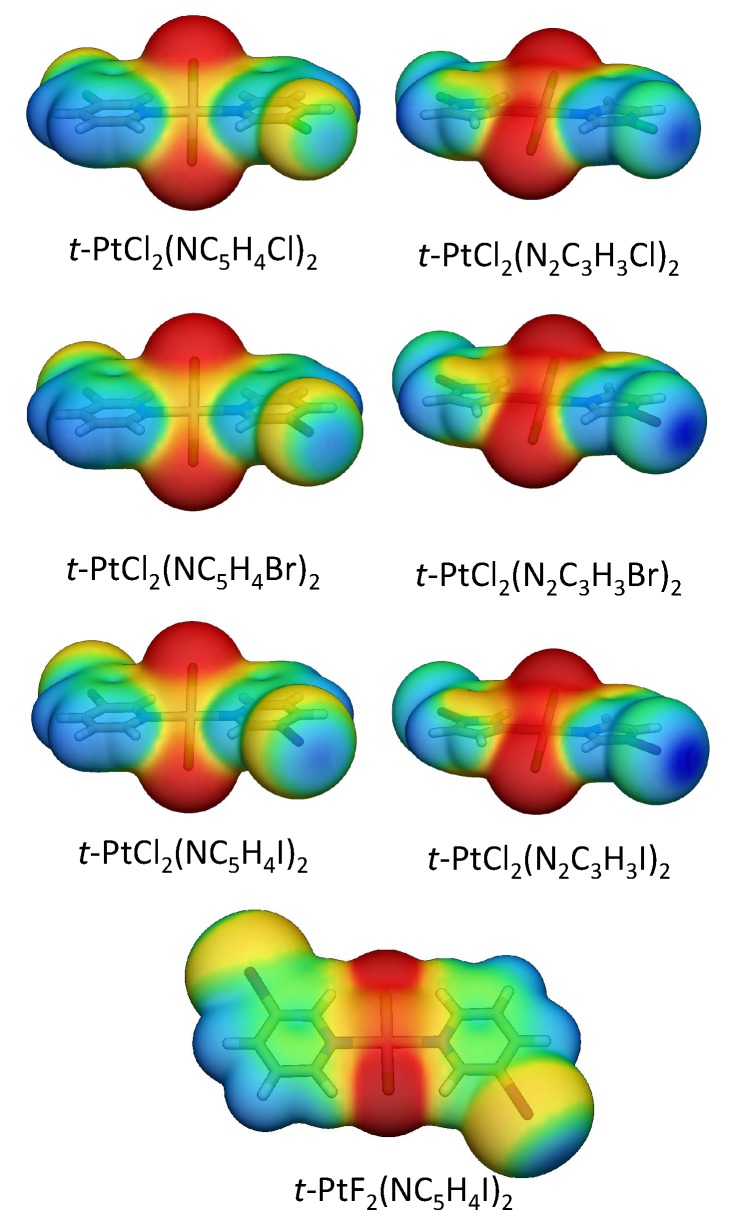
Electrostatic potential mapped onto the 0.001 a.u. electron density of selected monomers.

A more effective strategy to increase the XB strength is to substitute the halo-pyridine rings with halo-diazole rings, in which the halogen is bonded to a nitrogen. The larger electronegativity of the nitrogen polarizes the halogen atom more strongly, leading to a more positive electrostatic potential at the σ-hole and a low lying $\sigma^{*}$(NX) anti-bond orbital [16]. All halo-diazoles have more positive σ-holes than the comparable halo-pyridines, as shown in Figure 6. The bromo-diazole in **1.14** is able to form a stronger XB than any halo-pyridine derivative studied in this work (**1.1**–**1.12**) but has a ΔE of just 13.7 kcal/mol. Iodo-diazole derivative (**1.5**) has a distorted geometry, which allows the formation of both a strong halogen bond (BSO *n* = 0.352) of partial covalent character ($H_{b}$ = −0.017 Hartree/Å${}^{3}$) and a π-staking interaction between the iodine and a diazole ring with the largest binding energy found among all dimers studied in this work.

### 3.3. Comparison of Binding Modes 1 and 2

ΔE values for binding mode 1 dimers vary from 4.8 (**1.1**) to 27.5 kcal/mol (**1.15**). The major contribution to the stability of these complexes is XB, with a secondary participation of weak HB and nonspecific interactions (see Table 1). The ΔE variation in binding modes 2 dimers is considerably smaller, just 6 kcal/mol, ranging from ΔE(CCSD(T)) = 20.5 for **2.3** to 26.6 kcal/mol for **2.6**. This suggests that π-stacking interactions and weak HBs holding these dimers together are less sensitive to changes of the metal, halogen, or the aromatic ring coordinated to the metal. Therefore, in the following, only general trends of the most important interactions present in binding mode 2 dimers are discussed.

In binding mode 2 dimers involving halo-pyridine derivatives (**2.1**–**2.6**), HBs represent a minor contribution to the dimer stability (BSO *n*≈ 0.110). The most stabilizing contacts are electrostatic/dispersive interactions at the center of the complex, represented by a local stretching mode between the metal of one monomer and the midpoint of the XB metal of the other monomer (M…(M-X)), with a BSO *n* varying from 0.258 to 0.279 and the stacking of the pyridine ring (C_5_N)(C_5_N), which becomes predominantly a C–X…pyridine stacking as Cl is substituted with a higher homologue (X = Br or I), as evidenced by the bond paths and RDG of Figure 3 and BSO *n* values varying from 0.193 to 0.241. Dimer **2.7**, in which a CH of the iodo-piridine is substituted with N, has a more electronegative electrostatic potential at the inserted N close to the metal. Consequently, **2.7** adopts a skewed geometry to minimize electrostatic repulsion and to maximize electrostatic interactions. A nonspecific interaction between the Cl and a CH bond is evidenced by a bond path connecting Cl to C in **2.7** (BSO *n* = 0.232). The halo-diazole derivative (dimer **2.8**) does also adopt a skewed geometry to reduce electrostatic repulsion due to the negative electrostatic potential close to the metal at the π-region of the diazole ring (see Figure 6 and Figure S4). In this complex, both the metal and a Br atom form a nonspecific interaction with the halo-diazole rings of the opposite monomer.

### 3.4. Metal as a Halogen Bond Acceptor

Although all halogen-bonded crystal structures reported by Zordan et al. [25] have a halo-pyridine as the halogen donor and a Cl-metal ligand as halogen acceptor, several studies have shown that the d${}_{z^{2}}$ lone pair orbitals of Pt(II) and Pd(II) can participate directly in XB as halogen acceptor [35,75,76,77,78,79,80]. Therefore, we utilized an iodine molecule as a probe to investigate whether an XB with Pt can be formed as strong as the one formed with Cl.

**Figure 7:**
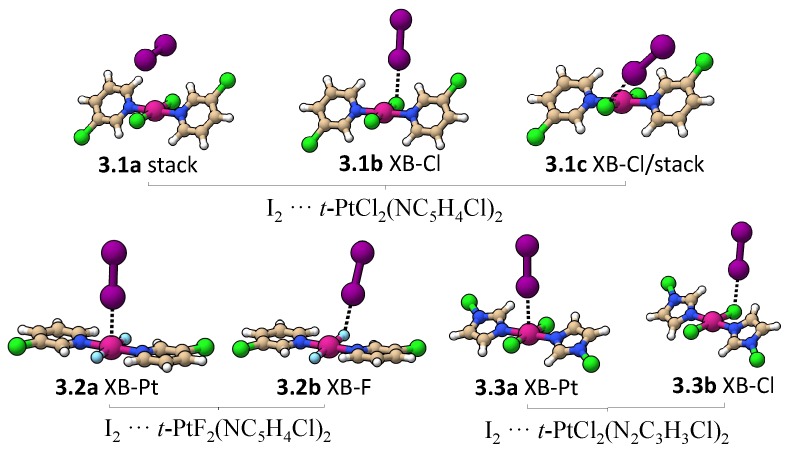
Minimum energy geometries involving an I_2_ and three different monomers.

Figure 7 shows all minima found for an I_2_ molecule interacting with trans-PtCl_2_(NC_5_H_4_Cl)_2_, trans-PtF_2_(NC_5_H_4_Cl)_2_, and trans-PtCl_2_(N_2_C_3_H_3_Cl)_2_. Table 3 provides interatomic distances, k${}^{a}$, BSO *n*, and ΔE values for these complexes. Three minima were found for I_2_···trans-PtCl_2_(NC_5_H_4_Cl)_2_ (**3.1a**, **3.1b**, and **3.1c**). In **3.1a**, the weakest bonded complex, the I_2_ molecule is oriented almost parallel to a Pt–Cl bond, resulting in a dispersive interaction similar to the one found between the Pt and a Pt–Cl bond in binding mode 2 (BSO *n* = 0.185). There is also a minor contribution from the electrostatic interaction of positively charged hydrogens with the lone pair π-density of I_2_ (BSO *n* = 0.065). If the I_2_ is moved closer to the Cl atom, two I…Cl-bonded minima are found (**3.1b** and **3.1c**), as shown in Figure 7. **3.1c** is more stable than **3.1b** due to the formation of a nonspecific interaction between the π-density of the iodine and the pyridine ring not present in **3.1b**. The absence of a I…Pt halogen-bonded minima in **3.1** complexes prompted us to investigate whether the complex is not formed because the Pt lone pairs are inaccessible to I_2_ due steric repulsion cause by the pyridine ring or due to the less negative electrostatic potential close to the metal compared to the Cl atom (see Figure 4). To test the first hypothesis, a constrained optimization was performed enforcing trans-PtCl_2_(NC_5_H_4_Cl)_2_ to be planar and positioning I_2_ collinear to Pt and orthogonal to the molecular plane. The optimized complex has a Pt…I distance of 3.310Å, which is just slightly longer than the Cl…I XB in **3.1b**. Local modes were computed after projecting out imaginary frequencies associated with pyridine ring rotation. The Pt…I interaction has a k${}^{a}$ of 0.128 mdyn/Å and a BSO *n* of 0.221, which is similar to the Cl…I XB strength. This confirms that it is the steric repulsion of the pyridine rings that preclude the formation of an XB with Pt in **3.1**. By substituting Cl with F, the steric repulsion between the ligands at Pt is reduced, resulting in a planar trans-PtF_2_(NC_5_H_4_Cl)_2_ molecule, in which the Pt atom becomes accessible for I_2_. In this case, two halogen-bonded minima were found; I_2_··· trans-PtF_2_(NC_5_H_4_Cl)_2_ (**3.2a** and **3.2b** in Figure 7). The stronger XB was found for **3.2b** (BSO *n* = 0.266 (**3.2a**); 0.304 (**3.2b**)) due to the lower electrostatic potential at the F atom, (see Figure 7). An XB with Pt is also found for I_2_ and halo-diazoles derivatives such as trans-PtCl_2_(N_2_C_3_H_3_Cl)_2_, being characterized by a lower electrostatic potential close to the metal (Figure 6) and less twisted rings (**3.3a**). Similar to **3.2** complexes, the I…Pt in **3.3a** is slightly weaker than the I…Cl in **3.3b**. Noteworthy is that the strength of the XB does also depend on the metal in this case. For example, Rogachev and coworkers found that I_2_ can coordinate more strongly to Co, Rh, and Ir compared to Ni, Pd, and Pt [143] and Oliveira and coworkers observed that, by varying the substituents at the halogen donor and acceptor, the XB metal can shift from weak electrostatic interaction (as observed in **3.2a** and **3.3a**) into a 3c-4e bond and even into a strong metal–halide bond [80].

## 4. Conclusions and Outlook

We investigated in this work 23 metal–halogen complexes trans-M(Y_2_)(NC_5_H_4_X-3)_2_ with M = Pd(II) and Pt(II); Y = F, Cl, Br; X = Cl, Br, I; and NC_5_H_4_X-3 = 3-halopyridine, derived from dimers, originally suggested by Zordan and coworkers [25]. Guided by the local mode analysis and complemented by energy and electron density analyses and electrostatic potential studies, we could for the first time quantify the various attractive contacts and their contribution to the dimer stability and clarify the special role of XB in these systems.

1. The largest contribution to the stability of the dimers is either due to XB or NS. HB plays only a secondary role as evidenced by the small BSO *n* values found for these compounds.
2. Systematic modeling and fine-tuning of the XB strength can only be achieved via a parameter that can quantify the strength of specific atom–atom interactions. As demonstrated in this work, the local stretching force constant and associated bond strength order BSO *n* are perfectly suited parameters for this purpose. For example, the unexpected increase of the binding energy by 3.4 kcal/mol observed by replacing the halogen at the metal Y = Cl (**1.4**) with iodine (**1.7**), which is a weaker halogen acceptor, can be unequivocally explained via the local mode force constants. The former has a stronger XB, but due to extra stabilization brought by dispersive interaction, the latter has a larger binding energy.
3. The best strategy for increasing the XB strength is to substitute the halo-pyridine ring with a halo-diazole ring. The larger polarization of the nitrogen bonds toward the halogen allows bromo-diazole derivatives to form stronger XBs than any of the iodo-pyridine systems investigated in this work. Noteworthy is that the iodo-diazole derivative has the strongest XB among the systems tested and that it is the only dimer in which the XB adopts partial covalent character (H${}_{b}<$ 0). All other dimers are held together by electrostatic and dispersive contacts.
4. The torsion of the pyridine ring caused by steric repulsion between the ligands at the metal atom makes the metal center $d_{z}{}^{2}$ lone pair orbital less accessible for XB than the lone pairs at the Cl ligands in trans-PtCl_2_(NC_5_H_4_Cl)_2_. If the Cl atoms at the metal are substituted by F or the pyridine ring is substituted by a smaller ring such as a diazole ring, a planar or quasi-planar structure is formed. In these systems, Pt can act as a halogen acceptor and can form an XB of similar strength as formed with a halogen.

Ongoing studies taking advantage of our recently developed local mode theory for periodic systems and crystal structures [136] are aimed at a better understanding of the subtle changes occurring in the crystal structures of these complexes upon variation of the metal and the halogen atom of the pyridine ring. These studies will also lead to new insights into the influence of crystal packing effects on the XB and HB strengths. We will also test if a stronger network of halogen bonds can be observed in halo-diazole derivatives.

## Figures and Tables

**Table 1:** Geometry, vibrational data, and topological properties of binding mode 1 dimers.

#	Dimers	Contact	Type	r	r(vdW)	k${}^{a}$	BSO *n*	$\rho_{b}$	$H_{b}$
**1.1**	(t-PdCl_2_(NC_5_H_4_Cl)_2_)_2_	Cl…Cl	XB	3.476	3.5	0.039	0.114	0.044	0.010
		H…Cl	HB	3.051	3.0	0.042	0.119	0.028	0.006
**1.2**	(t-PtCl_2_(NC_5_H_4_Cl)_2_)_2_	Cl…Cl	XB	3.457	3.5	0.051	0.133	0.046	0.010
		H…Cl	HB	3.017	3.0	0.030	0.099	0.030	0.006
**1.3**	(t-PtCl_2_(NC_5_H_4_Br)_2_)_2_	Br…Cl	XB	3.336	3.6	0.090	0.182	0.071	0.009
		H…Br	HB	3.027	3.1	0.045	0.124	0.039	0.006
**1.4**	(t-PtCl_2_(NC_5_H_4_I)_2_)_2_	I…Cl	XB	3.353	3.7	0.108	0.201	0.089	0.008
		H…I	HB	3.143	3.2	0.060	0.145	0.044	0.005
**1.5**	(t-PtF_2_(NC_5_H_4_I)_2_)_2_	I…F	XB	2.893	3.5	0.111	0.204	0.111	0.013
		H…I	HB	3.068	3.2	0.085	0.176	0.051	0.006
**1.6**	(t-PtBr_2_(NC_5_H_4_I)_2_)_2_	I…Br	XB	3.654	3.8	0.057	0.141	0.065	0.005
		I…(H-C)	NS	3.761	3.7	0.094	0.186	0.039	0.005
**1.7**	(t-PtI_2_(NC_5_H_4_I)_2_)_2_	I…I	XB	3.812	4.0	0.062	0.148	0.064	0.004
		I…(H-C)	NS	3.799	3.7	0.086	0.177	0.039	0.005
**1.8a**	(t-PtCl_2_(N_2_C_4_H_3_I)_2_)_2_	I…Cl	XB	3.304	3.7	0.127	0.220	0.099	0.007
		I…(H-C)	NS	3.763	3.7	0.099	0.192	0.042	0.006
**1.8b**	(t-PtCl_2_(N_2_C_4_H_3_I)_2_)_2_	I…N	XB	3.205	3.5	0.083	0.174	0.081	0.010
		I…(Cl-Pt)	NS	3.792	3.7	0.118	0.211	0.042	0.006
**1.9**	(t-PtCl_2_(N_2_C_4_H_3_I)_2_)_2_	I…Cl	XB	3.344	3.7	0.104	0.197	0.082	0.008
		H…I	HB	3.131	3.2	0.056	0.140	0.045	0.005
**1.10**	(t-PtCl_2_(N_2_C_4_H_3_I)_2_)_2_	I…Cl	XB	3.393	3.7	0.102	0.195	0.082	0.008
		H…I	HB	3.135	3.2	0.055	0.138	0.044	0.005
**1.11**	(t-PtCl_2_(N_2_C_4_H_3_I)_2_)_2_	I…Cl	XB	3.300	3.7	0.156	0.247	0.097	0.008
**1.12**	(t-PtCl_2_(N_3_C_3_H_2_I)_2_)_2_	I…Cl	XB	3.280	3.7	0.142	0.234	0.104	0.007
		I…(CH)	NS	3.543	3.2	0.083	0.174	0.041	0.006
**1.13**	(t-PtCl_2_(N_2_C_3_H_3_Cl)_2_)_2_	Cl…Cl	XB	3.144	3.5	0.068	0.156	0.085	0.013
		Cl…H	HB	2.846	3.0	0.025	0.089	0.048	0.009
**1.14**	(t-PtCl_2_(N_2_C_3_H_3_Br)_2_)_2_	Br…Cl	XB	2.894	3.6	0.176	0.264	0.171	0.001
		H…Br	HB	2.917	3.1	0.053	0.135	0.061	0.008
**1.15**	(t-PtCl_2_(N_2_C_3_H_3_I)_2_)_2_	I…Cl	XB	2.890	3.7	0.295	0.352	0.219	−0.017
		I…(CN)	NS	3.568	3.7	0.195	0.279	0.053	0.006

${}^{a}$Atom–atom specific interactions are hydrogen bonds (HBs) or halogen bonds (XBs). Nonspecific (NS) interaction involves the midpoint of a bond (in brackets). r is the distance in Å between two atoms or an atom and bond midpoints, and r(vdW) is the sum of Bondi radii of the closest two atoms. Local stretching force constant (k${}^{a}$) in mdyn/Å, bond strength order (BSO), density at the BCP ($\rho_{b}$) in e/Å${}^{3}$, and energy density at the BCP in Hartree/Å${}^{3}$ are also shown.

**Table 2:** Binding energies and enthalpies of all dimers.

#	Dimers	ΔE(DFT)	ΔE(DFT-CP)	ΔE(CCSD(T))	ΔH
**1.1**	(t-PdCl_2_(NC_5_H_4_Cl)_2_)_2_	3.6	3.4	4.8	3.8
**1.2**	(t-PtCl_2_(NC_5_H_4_Cl)_2_)_2_	3.9	3.7	5.0	3.8
**1.3**	(t-PtCl_2_(NC_5_H_4_Br)_2_)_2_	7.0	6.8	6.7	5.4
**1.4**	(t-PtCl_2_(NC_5_H_4_I)_2_)_2_	10.8	10.5	10.1	9.0
**1.5**	(t-PtF_2_(NC_5_H_4_I)_2_)_2_	11.9	11.6	12.4	11.2
**1.6**	(t-PtBr_2_(NC_5_H_4_I)_2_)_2_	12.9	12.6	13.7	12.5
**1.7**	(t-PtI_2_(NC_5_H_4_I)_2_)_2_	13.3	13.0	13.5	12.3
**1.8a**	(t-PtCl_2_(N_2_C_4_H_3_I)_2_)_2_	17.3	17.0	17.2	16.0
**1.8b**	(t-PtCl_2_(N_2_C_4_H_3_I)_2_)_2_	15.9	15.5	16.3	15.0
**1.9**	(t-PtCl_2_(N_2_C_4_H_3_I)_2_)_2_	11.1	10.8	10.5	9.2
**1.10**	(t-PtCl_2_(N_2_C_4_H_3_I)_2_)_2_	9.8	9.5	9.7	8.3
**1.11**	(t-PtCl_2_(N_2_C_4_H_3_I)_2_)_2_	13.4	13.1	13.5	12.3
**1.12**	(t-PtCl_2_(N_3_C_3_H_2_I)_2_)_2_	18.3	17.9	18.0	16.7
**1.13**	(t-PtCl_2_(N_2_C_3_H_3_Cl)_2_)_2_	8.2	7.9	8.9	7.5
**1.14**	(t-PtCl_2_(N_2_C_3_H_3_Br)_2_)_2_	15.6	15.3	13.7	12.2
**1.15**	(t-PtCl_2_(N_2_C_3_H_3_I)_2_)_2_	27.4	27.0	27.5	26.5
**2.1**	(t-PdCl_2_(NC_5_H_4_Cl)_2_)_2_	20.6	19.5	24.2	22.8
**2.2**	(t-PtCl_2_(NC_5_H_4_Cl)_2_)_2_	22.6	21.6	23.1	21.9
**2.3**	(t-PtCl_2_(NC_5_H_4_F)_2_)_2_	21.5	20.3	20.5	19.7
**2.4**	(t-PtCl_2_(NC_5_H_4_Br)_2_)_2_	23.7	22.7	24.3	23.2
**2.5**	(t-PtCl_2_(NC_5_H_4_I)_2_)_2_	24.9	24.0	25.0	23.9
**2.6**	(t-PtBr_2_(NC_5_H_4_I)_2_)_2_	26.3	25.5	26.6	25.4
**2.7**	(t-PtCl_2_(N_2_C_4_H_3_I)_2_)_2_	22.5	21.6	22.6	21.4
**2.8**	(t-PtCl_2_(N_2_C_3_H_3_Br)_2_)_2_	22.3	21.4	22.2	20.7

Values are in kcal/mol. Binding energies (ΔE) are computed at ωB97XD/jun-cc-pVTZ(-PP) without CP to the basis set superposition error (ΔE(DFT)) and with CP correction (ΔE(DFT-CP)). DLPNO-CCSD(T)/ma-def2-TZVP binding energies (ΔE(CCSD(T)) and enthalpies ΔH. Zero-point energy, and thermal correction are obtained at the ωB97XD level.

**Table 3:** Interatomic distances, force constant, and binding energy of iodine–metal complexes.

#	System	Type	r	k${}^{a}$	BSO *n*	ΔE
**3.1a**	I_2_…t-PtCl_2_(NC_5_H_4_Cl)_2_	stack	4.065	0.093	0.185	6.7
**3.1b**		XB-Cl	3.118	0.144	0.236	8.5
**3.1c**		XB-Cl/stack	3.188	0.149	0.241	9.3
**3.2a**	I_2_…t-PtF_2_(NC_5_H_4_Cl)_2_	XB-Pt	3.152	0.179	0.266	8.7
**3.2b**		XB-F	2.658	0.227	0.304	9.5
**3.3a**	I_2_…t-PtCl_2_(N_2_C_3_H_3_Cl)_2_	XB-Pt	3.259	0.134	0.227	8.5
**3.3b**		XB-Cl	3.098	0.152	0.243	8.7

Distance in Å, $k^{a}$ in mdyn/Å and ΔE(DFT) in values in kcal/mol.

## Supplementary Materials

The following are available at https://www.mdpi.com/1996-1944/13/1/55/s1, Figure S1 and Figure S2: bar diagrams comparing the acendent ordering of ΔE values computed at DFT and DLPNO-CCSD(T), Figure S3: ΔE(DFT) *versus* k${}^{a}$(XB), Figure S4: RDG and bond paths of selected systems, Figure S5: Relative energy associated to the torsion of a pyridine ring, Figure S6: Selected atomic orbitals, Table S1: Geometry and vibrational and topological properties of binding modes 2.

## References

[1] Desiraju G.R., Ho P.S., Kloo L., Legon A.C., Marquardt R., Metrangolo P., Politzer P., Resnati G., Rissanen K. (2013). Definition of the halogen bond (IUPAC Recommendations 2013). Pure Appl. Chem..

[2] Varadwaj P.R., Varadwaj A., Marques H.M. (2019). Halogen Bonding: A Halogen-Centered Noncovalent Interaction Yet to Be Understood. Inorganics.

[3] Guthrie F. (1863). Xxviii.—On the Iodide of Iodammonium. J. Chem. Soc..

[4] Mulliken R.S. (1950). Structures of Complexes Formed by Halogen Molecules with Aromatic and with Oxygenated Solvents. J. Am. Chem. Soc..

[5] Mulliken R.S. (1952). Molecular Compounds and their Spectra. II. J. Am. Chem. Soc..

[6] Mulliken R.S. (1952). Molecular Compounds and their Spectra. III. The Interaction of Electron Donors and Acceptors. J. Phys. Chem..

[7] Hassel O., Hvoslef J., Vihovde E.H., Sorensen N.A. (1954). The Structure of Bromine 1,4-Dioxanate. Acta Chem. Scand..

[8] Cavallo G., Metrangolo P., Milani R., Pilati T., Priimagi A., Resnati G., Terraneo G. (2016). The Halogen Bond. Chem. Rev..

[9] Riel A.M.S., Rowe R.K., Ho E.N., Carlsson A.C.C., Rappe A.K., Berryman O.B., Ho P.S. (2019). Hydrogen Bond Enhanced Halogen Bonds: A Synergistic Interaction in Chemistry and Biochemistry. Acc. Chem. Res..

[10] Brammer L. (2017). Halogen bonding, chalcogen bonding, pnictogen bonding, tetrel bonding: Origins, current status and discussion. Faraday Discuss.

[11] Gilday L., Robinson S., Barendt T., Langton M., Mullaney B., Beer P. (2015). Halogen Bonding in Supramolecular Chemistry. Chem. Rev..

[12] Lisac K., Topic F., Arhangelskis M., Julien S.C.P.A., Nickels C.W., Morris A.J., Friscic T., Cincic D. (2019). Halogen-bonded cocrystallization with phosphorus, arsenic and antimony acceptors. Nat. Commun..

[13] Metrangolo P., Resnati G., Pilati T., Liantonio R., Meyer F. (2007). Engineering Functional Materials by Halogen Bonding. J. Polym. Sci. Part A Polym. Chem..

[14] Li B., Zang S.Q., Wang L.Y., Mak T.C. (2016). Halogen bonding: A powerful, emerging tool for constructing high-dimensional metal-containing supramolecular networks. Coord. Chem. Rev..

[15] Oliveira V., Kraka E., Cremer D. (2016). The Intrinsic Strength of the Halogen Bond: Electrostatic and Covalent Contributions Described by Coupled Cluster Theory. Phys. Chem. Chem. Phys..

[16] Oliveira V., Kraka E., Cremer D. (2016). Quantitative Assessment of Halogen Bonding Utilizing Vibrational Spectroscopy. Inorg. Chem..

[17] Oliveira V., Kraka E. (2017). Systematic Coupled Cluster Study of Noncovalent Interactions Involving Halogens, Chalcogens, and Pnicogens. J. Phys. Chem. A.

[18] Freindorf M., Kraka E., Cremer D. (2012). A Comprehensive Analysis of Hydrogen Bond Interactions Based on Local Vibrational Modes. Int. J. Quant. Chem..

[19] Stone A.J. (2013). Are Halogen Bonded Structures Electrostatically Driven?. J. Am. Chem. Soc..

[20] Hill J.G., Legon A.C. (2015). On the directionality and non-linearity of halogen and hydrogen bonds. Phys. Chem. Chem. Phys..

[21] Legon A.C. (2010). The halogen bond: An interim perspective. Phys. Chem. Chem. Phys..

[22] Clark T., Hennemann M., Murray J., Politzer P. (2007). Halogen bonding: The *σ*-hole. J. Mol. Model..

[23] Clark T. (2017). Halogen bonds and *σ*-holes. Faraday Discuss..

[24] Politzer P., Murray J., Clark T., Resnati G. (2017). The *σ*-hole revisited. Phys. Chem. Chem. Phys..

[25] Zordan F., Brammer L., Sherwood P. (2005). Supramolecular Chemistry of Halogens: Complementary Features of Inorganic (M-X) and Organic (C-X’) Halogens Applied to M-X…X’-C Halogen Bond Formation. J. Am. Chem. Soc..

[26] Bertani R., Sgarbossa P., Venzo A., Lelj F., Amati M., Resnati G., Pilati T., Metrangolo P., Terraneo G. (2010). Halogen bonding in metal-organic-supramolecular networks. Coord. Chem. Rev..

[27] Brammer L., Mínguez Espallargas G., Libri S. (2008). Combining metals with halogen bonds. CrystEngComm.

[28] Van Beek J.A.M., van Koten G., Smeets W.J.J., Spek A.L. (1986). Model for the Initial Stage in the Oxidative Addition of I_2_ to Organoplatinum(II) Compounds. X-ray Structure of Square-Pyramidal [Pt^*II*^C_6_H_3_(CH_2_NMe_2_)_2_-o,o^′^(*η*^1^ − *I*_2_)] Containing a Linear Pt-I-I Arrangement. J. Am. Chem. Soc..

[29] Minguez Espallargas G., Brammer L., Allan D.R., Pulham C.R., Robertson N., Warren J.E. (2008). Noncovalent Interactions under Extreme Conditions: High-Pressure and Low-Temperature Diffraction Studies of the Isostructural Metal-Organic Networks (4-Chloropyridinium)_2_[CoX_4_] (X = Cl, Br). J. Am. Chem. Soc..

[30] Libri S., Jasim N.A., Perutz R.N., Brammer L. (2008). Metal Fluorides Form Strong Hydrogen Bonds and Halogen Bonds: Measuring Interaction Enthalpies and Entropies in Solution. J. Am. Chem. Soc..

[31] Mingues Espallargas G., Zordan F., Arroyo L., Adams H., Shankland K., van Streek J., Brammer L. (2009). Rational Modification of the Hierarchy of Intermolecular Interactions in Molecular Crystal Structures by Using Tunable Halogen Bonds. Chem. Eur. J..

[32] Thangavadivale V., Aguiar P.M., Jasim N.A., Pike S.J., Smith D.A., Whitwood A.C., Brammer L., Perutz R.N. (2018). Self-complementary nickel halides enable multifaceted comparisons of intermolecular halogen bonds: fluoride ligands vs. other halides. Chem. Sci..

[33] Von Essen C., Rissanen K., Puttreddy R. (2019). Halogen Bonds in 2,5-Dihalopyridine-Copper(I) Halide Coordination Polymers. Materials.

[34] Awwadi F.F., Turnbull M.M., Alwahsh M.I., Haddad S.F. (2018). May halogen bonding interactions compete with Cu…Cl semi-coordinate bonds? Structural, magnetic and theoretical studies of two polymorphs of trans-bis(5-bromo-2-chloro pyridine)dichlorocopper(ii) and trans-bis(2,5-dichloropyridine) dichlorocopper(ii). New J. Chem..

[35] Baykov S.V., Dabranskaya U., Ivanov D.M., Novikov A.S., Boyarskiy V.P. (2018). Pt/Pd and I/Br Isostructural Exchange Provides Formation of C-I…Pd, C-Br…Pt, and C-Br…Pd Metal-Involving Halogen Bonding. Cryst. Growth Des..

[36] Scheiner S. (2019). On the capability of metal-halogen groups to participate in halogen bonds. CrystEngComm.

[37] Mahmudov K.T., Gurbanov A.V., Guseinov F.I., da Silva M.F.C.G. (2019). Noncovalent interactions in metal complex catalysis. Coord. Chem. Rev..

[38] Corpinot M.K., Bucar D.K. (2019). A Practical Guide to the Design of Molecular Crystals. Cryst. Growth Des..

[39] Aakeröy C.B., Spartz C.L., Dembowski S., Dwyre S., Desper J. (2015). A systematic structural study of halogen bonding *versus* hydrogen bonding within competitive supramolecular systems. IUCrJ.

[40] Wu D., Dong C., Zhan H., Du X.W. (2018). Bond-Energy-Integrated Descriptor for Oxygen Electrocatalysis of Transition Metal Oxides. J. Phys. Chem. Lett..

[41] Lai W., Li C., Chen H., Shaik S. (2012). Hydrogen-abstraction reactivity patterns from A to Y: The valence bond way. Angew. Chem. Int. Ed..

[42] Stasyuk O.A., Sedlak R., Guerra C.F., Hobza P. (2018). Comparison of the DFT-SAPT and canonical EDA Schemes for the energy decomposition of various types of noncovalent interactions. J. Chem. Theory Comput..

[43] Levine D.S., Head-Gordon M. (2017). Energy decomposition analysis of single bonds within Kohn-Sham density functional theory. Proc. Natl. Acad. Sci. USA.

[44] Andrés J., Ayers P.W., Boto R.A., Carbó-Dorca R., Chermette H., Cioslowski J., Contreras-García J., Cooper D.L., Frenking G., Gatti C. (2019). Nine questions on energy decomposition analysis. J. Comput. Chem..

[45] Cremer D., Kraka E. (2010). From Molecular Vibrations to Bonding, Chemical Reactions, and Reaction Mechanism. Curr. Org. Chem..

[46] Zou W., Kalescky R., Kraka E., Cremer D. (2012). Relating Normal Vibrational Modes to Local Vibrational Modes with the Help of an Adiabatic Connection Scheme. J. Chem. Phys..

[47] Murray J., Politzer P. (2017). Molecular electrostatic potentials and noncovalent interactions. WIREs Comput. Mol. Sci..

[48] Bader R.F.W. (1990). Atoms in Molecules—A Quantum Theory.

[49] Li S., Xu T., van Mourik T., Frc̎htl H., Kirk S.R., Jenkins S. (2019). Halogen and Hydrogen Bonding in Halogenabenzene/NH_3_ Complexes Compared Using Next-Generation QTAIM. Molecules.

[50] Chen Z., Wang G., Xu Z., Wang J., Yu Y., Cai T., Shao Q., Shi J., Zhu W. (2016). How Do Distance and Solvent Affect Halogen Bonding Involving Negatively Charged Donors?. J. Phys. Chem. B.

[51] Bader R.F.W. (1998). A Bond Path: A Universal Indicator of Bonded Interactions. J. Phys. Chem. A.

[52] Bader R.F.W. (2009). Bond Paths Are Not Chemical Bonds. J. Phys. Chem. A.

[53] Jablonski M. (2018). Bond paths between distant atoms do not necessarily indicate dominant interactions. J. Comput. Chem..

[54] Jablonski M. (2019). On the Uselessness of Bond Paths Linking Distant Atoms and on the Violation of the Concept of Privileged Exchange Channels. ChemistryOpen.

[55] Luo Y.R. (2007). Comprehensive Handbook of Chemical Bond Energies.

[56] Moltved K.A., Kepp K.P. (2018). Chemical Bond Energies of 3d Transition Metals Studied by Density Functional Theory. J. Chem. Theory Comput..

[57] Kosar N., Ayub K., Gilani M.A., Mahmood T. (2019). Benchmark DFT studies on C−CN homolytic cleavage and screening the substitution effect on bond dissociation energy. J. Mol. Model..

[58] Morse M.D. (2018). Predissociation measurements of bond dissociation energies. Acc. Chem. Res..

[59] Fang Z., Vasiliu M., Peterson K.A., Dixon D.A. (2017). Prediction of bond dissociation energies/heats of formation for diatomic transition metal compounds: CCSD(T) works. J. Chem. Theory Comput..

[60] Kuznetsov M.L. (2019). Relationships between Interaction Energy and Electron Density Properties for Homo Halogen Bonds of the [(A)_n_Y-X…X-Z(B)_m_] Type (X = Cl, Br, I). Molecules.

[61] Kalescky R., Kraka E., Cremer D. (2014). Are Carbon-Halogen Double and Triple Bonds Possible?. Int. J. Quant. Chem..

[62] Kalescky R., Zou W., Kraka E., Cremer D. (2014). Quantitative Assessment of the Multiplicity of Carbon-Halogen Bonds: Carbenium and Halonium Ions with F, Cl, Br, and I. J. Phys. Chem. A.

[63] Setiawan D., Sethio D., Cremer D., Kraka E. (2018). From Strong to Weak NF Bonds: On the Design of a New Class of Fluorinating Agents. Phys. Chem. Chem. Phys..

[64] Sethio D., Oliveira V., Kraka E. (2018). Quantitative Assessment of Tetrel Bonding Utilizing Vibrational Spectroscopy. Molecules.

[65] Kraka E., Cremer D. (2009). Characterization of CF Bonds with Multiple-Bond Character: Bond Lengths, Stretching Force Constants, and Bond Dissociation Energies. ChemPhysChem.

[66] Cremer D., Larsson J.A., Kraka E., Parkanyi C. (1998). New Developments in the Analysis of Vibrational Spectra on the Use of Adiabatic Internal Vibrational Modes. Theoretical and Computational Chemistry.

[67] Konkoli Z., Larsson J.A., Cremer D. (1998). A New Way of Analyzing Vibrational Spectra. II. Comparison of Internal Mode Frequencies. Int. J. Quant. Chem..

[68] Konkoli Z., Cremer D. (1998). A New Way of Analyzing Vibrational Spectra. III. Characterization of Normal Vibrational Modes in terms of Internal Vibrational Modes. Int. J. Quant. Chem..

[69] Kaupp M., Danovich D., Shaik S. (2017). Chemistry is about energy and its changes: A critique of bond-length/bond-strength correlations. Coord. Chem. Rev..

[70] Wilson E.B., Decius J.C., Cross P.C. (1955). Molecular Vibrations. The Theory of Infrared and Raman Vibrational Spectra.

[71] Thakuria R., Nath N.K., Saha B.K. (2019). The Nature and Applications of *π*-*p**i* Interactions: A Perspective. Cryst. Growth Des..

[72] Wang H., Wang W., Jin W.J. (2016). *σ*Hole Bond vs *π*-Hole Bond: A Comparison Based on Halogen Bond. Chem. Rev..

[73] Konkoli Z., Cremer D. (1998). A New Way of Analyzing Vibrational Spectra. I. Derivation of Adiabatic Internal Modes. Int. J. Quant. Chem..

[74] Konkoli Z., Larsson J.A., Cremer D. (1998). A New Way of Analyzing Vibrational Spectra. IV. Application and Testing of Adiabatic Modes within the Concept of the Characterization of Normal Modes. Int. J. Quant. Chem..

[75] Rogachev A.Y., Hoffmann R. (2013). Iodine (I_2_) as a Janus-Faced Ligand in Organometallics. J. Am. Chem. Soc..

[76] Lu B., Zhang X., Meng L., Zeng Y. (2016). The Pt (II)…Cl Interactions: Nature and Strength. ChemistrySelect.

[77] Gossage R.A., Ryabov A.D., Spek A.L., Stufkens D.J., van Beek J.A.M., van Eldik R., van Koten G. (1999). Models for the Initial Stages of Oxidative Addition. Synthesis, Characterization, and Mechanistic Investigation of *η*^1^-I_2_ Organometallic "Pincer" Complexes of Platinum. X-ray Crystal Structures of [PtI(C_6_H_3_CH_2_NMe_2_2-2,6)(*η*^1^-I_2_)] and exo-meso-[Pt(*η*^1^-I_3_)(*η*^1^-I_2_)(C_6_H_3_CH_2_N(t-Bu)Me2-2,6)]. J. Am. Chem. Soc..

[78] Ivanov D.M., Novikov A.S., Ananyev I.V., Kirina Y.V., Kukushkin V.Y. (2016). Halogen bonding between metal centers and halocarbons. Chem. Commun..

[79] Bikbaeva Z.M., Ivanov D.M., Novikov A.S., Ananyev I.V., Bokach N.A., Kukushkin V.Y. (2017). Electrophilic-Nucleophilic Dualism of Nickel(II) toward Ni…I Noncovalent Interactions: Semicoordination of Iodine Centers via Electron Belt and Halogen Bonding via *σ*-Hole. Inorg. Chem..

[80] Oliveira V., Cremer D. (2017). Transition from Metal-Ligand Bonding to Halogen Bonding Involving a Metal as Halogen Acceptor: A Study of Cu, Ag, Au, Pt, and Hg Complexes. Chem. Phys. Lett..

[81] Chai J.D., Head-Gordon M. (2008). Long-range corrected hybrid density functionals with damped atom-atom dispersion corrections. Phys. Chem. Chem. Phys..

[82] Grimme S. (2006). Semiempirical hybrid density functional with perturbative second-order correlation. J. Chem. Phys..

[83] Mardirossian N., Head-Gordon M. (2017). Thirty years of density functional theory in computational chemistry: An overview and extensive assessment of 200 density functionals. Mol. Phys..

[84] Papajak E., Zheng J., Xu X., Leverentz H.R., Truhlar D.G. (2011). Perspectives on Basis Sets Beautiful: Seasonal Plantings of Diffuse Basis Functions. J. Chem. Theory Comput..

[85] Dunning T.H. (1989). Gaussian basis sets for use in correlated molecular calculations. I. The atoms boron through neon and hydrogen. J. Chem. Phys..

[86] Peterson K.A., Figgen D., Dolg M., Stoll H. (2007). Energy-consistent relativistic pseudopotentials and correlation consistent basis sets for the 4d elements Y-Pd. J. Chem. Phys..

[87] Figgen D., Peterson K.A., Dolg M., Stoll H. (2009). Energy-consistent pseudopotentials and correlation consistent basis sets for the 5d elements Hf-Pt. J. Chem. Phys..

[88] Peterson K.A., Figgen D., Goll E., Stoll H., Dolg M. (2003). Systematically convergent basis sets with relativistic pseudopotentials. II. Small-core pseudopotentials and correlation consistent basis sets for the post-d group 16–18 elements. J. Chem. Phys..

[89] Peterson K.A., Shepler B.C., Figgen D., Stoll H. (2006). On the Spectroscopic and Thermochemical Properties of ClO, BrO, IO, and Their Anions. J. Phys. Chem. A.

[90] Gräfenstein J., Izotov D., Cremer D. (2007). Avoiding Singularity Problems Associated with Meta-GGA (Generalized Gradient Approximation) Exchange and Correlation Functionals Containing the Kinetic Energy Density. J. Chem. Phys..

[91] Boys S., Bernardi F. (1970). The calculation of small molecular interactions by the differences of separate total energies. Some procedures with reduced errors. Mol. Phys..

[92] Sheng X.W., Mentel L., Gritsenko O.V., Baerends E.J. (2011). Counterpoise correction is not useful for short and Van der Waals distances but may be useful at long range. J. Comput. Chem..

[93] Raghavachari K., Trucks G.W., Pople J.A., Head-Gordon M. (1989). A fifth-order perturbation comparison of electron correlation theories. Chem. Phys. Lett..

[94] Guo Y., Riplinger C., Becker U., Liakos D.G., Minenkov Y., Cavallo L., Neese F. (2018). Communication: An improved linear scaling perturbative triples correction for the domain based local pair-natural orbital based singles and doubles coupled cluster method [DLPNO-CCSD(T)]. J. Chem. Phys..

[95] Weigend F., Ahlrichs R. (2005). Balanced basis sets of split valence, triple zeta valence and quadruple zeta valence quality for H to Rn: Design and assessment of accuracy. Phys. Chem. Chem. Phys..

[96] Zheng J., Xu X., Truhlar D.G. (2011). Minimally augmented Karlsruhe basis sets. Theor. Chem. Acc..

[97] Liakos D.G., Sparta M., Kesharwani M.K., Martin J.M.L., Neese F. (2015). Exploring the Accuracy Limits of Local Pair Natural Orbital Coupled-Cluster Theory. J. Chem. Theory Comput.

[98] Hellweg A., Hättig C., Höfener S., Klopper W. (2007). Optimized accurate auxiliary basis sets for RI-MP2 and RI-CC2 calculations for the atoms Rb to Rn. Theor. Chem. Acc..

[99] Liakos D.G., Neese F. (2015). Is It Possible To Obtain Coupled Cluster Quality Energies at near Density Functional Theory Cost? Domain-Based Local Pair Natural Orbital Coupled Cluster vs Modern Density Functional Theory. J. Chem. Theory Comput.

[100] Cremer D., Kraka E. (1984). Chemical Bonds without Bonding Electron Density? Does the Difference Electron-Density Analysis Suffice for a Description of the Chemical Bond?. Angew. Chem. Int. Ed..

[101] Cremer D., Kraka E. (1984). A Description of the Chemical Bond in Terms of Local Properties of Electron Density and Energy. Croat. Chem. Acta.

[102] Oliveira V., Cremer D., Kraka E. (2017). The Many Facets of Chalcogen Bonding: Described by Vibrational Spectroscopy. J. Phys. Chem. A.

[103] Kraka E., Cremer D., Maksic Z.B. (1990). Chemical Implication of Local Features of the Electron Density Distribution. Theoretical Models of Chemical Bonding. The Concept of the Chemical Bond.

[104] Contreras-Garcia J., Johnson E.R., Keinan S., Chaudret R., Piquemal J.P., Beratan D.N., Yang W. (2011). NCIPLOT: A Program for Plotting Noncovalent Interaction Regions. J. Chem. Theory Comput..

[105] Badger R.M. (1934). A Relation between Internuclear Distances and Bond Force Constants. J. Chem. Phys..

[106] Gordy W. (1946). A Relation between Bond Force Constants, Bond Orders, Bond Lengths, and the Electronegativities of the Bonded Atoms. J. Chem. Phys..

[107] Legon A.C. (1999). Prereactive Complexes of Dihalogens XY with Lewis Bases B in the Gas Phase: A Systematic Case for the Halogen Analogue B*c**d**o**t**s*XY of the Hydrogen Bond B…HX. Angew. Chem. Int. Ed..

[108] Kraka E., Larsson J.A., Cremer D., Grunenberg J. (2010). Generalization of the Badger Rule Based on the Use of Adiabatic Vibrational Modes. Computational Spectroscopy.

[109] Herzberg G. (1945). Molecular Spectra and Molecular Structure, II. Infrared and Raman Spectra of Polyatomic Molecules.

[110] Wilson E.B. (1939). A Method of Obtaining the Expanded Secular Equation for the Vibration Frequencies of a Molecule. J. Chem. Phys..

[111] Woodward L.A. (1972). Introduction to the Theory of Molecular Vibrations and Vibrational Spectroscopy.

[112] Califano S. (1976). Vibrational States.

[113] Decius J. (1963). Compliance matrix and molecular vibrations. J. Chem. Phys..

[114] Zou W., Kalescky R., Kraka E., Cremer D. (2012). Relating Normal Vibrational Modes to Local Vibrational Modes: Benzene and Naphthalene. J. Mol. Model..

[115] Zou W., Cremer D. (2014). Properties of Local Vibrational Modes: The Infrared Intensity. Theor. Chem. Acc..

[116] Kalescky R., Kraka E., Cremer D. (2013). Identification of the Strongest Bonds in Chemistry. J. Phys. Chem. A.

[117] Kraka E., Setiawan D., Cremer D. (2015). Re-Evaluation of the Bond Length-Bond Strength Rule: The Stronger Bond Is not Always the Shorter Bond. J. Comp. Chem..

[118] Zou W., Cremer D. (2016). C_2_ in a Box: Determining its Intrinsic Bond Strength for the X^1^Σ^+^_g_ Ground State. Chem. Eur. J..

[119] Sethio D., Daku L.M.L., Hagemann H., Kraka E. (2019). Quantitative Assessment of B−B−B, B−H_b_−B, and B−H_t_ Bonds: From BH_3_ to B_12_H_12_^2−^. ChemPhysChem.

[120] Yannacone S., Oliveira V., Verma N., Kraka E. (2019). A Continuum from Halogen Bonds to Covalent Bonds: Where Do *λ*^3^ Iodanes Fit?. Inorganics.

[121] Setiawan D., Kraka E., Cremer D. (2015). Hidden Bond Anomalies: The Peculiar Case of the Fluorinated Amine Chalcogenides. J. Phys. Chem. A.

[122] Setiawan D., Kraka E., Cremer D. (2014). Strength of the Pnicogen Bond in Complexes Involving Group VA Elements N, P, and As. J. Phys. Chem. A.

[123] Setiawan D., Kraka E., Cremer D. (2014). Description of Pnicogen Bonding with the help of Vibrational Spectroscopy-The Missing Link Between Theory and Experiment. Chem. Phys. Letters.

[124] Setiawan D., Cremer D. (2016). Super-Pnicogen Bonding in the Radical Anion of the Fluorophosphine Dimer. Chem. Phys. Lett..

[125] Kalescky R., Zou W., Kraka E., Cremer D. (2012). Local Vibrational Modes of the Water Dimer - Comparison of Theory and Experiment. Chem. Phys. Lett..

[126] Kalescky R., Kraka E., Cremer D. (2013). Local Vibrational Modes of the Formic Acid Dimer—The Strength of the Double H-Bond. Mol. Phys..

[127] Tao Y., Zou W., Jia J., Li W., Cremer D. (2016). Different Ways of Hydrogen Bonding in Water—Why Does Warm Water Freeze Faster than Cold Water?. J. Chem. Theory Comput..

[128] Tao Y., Zou W., Kraka E. (2017). Strengthening of Hydrogen Bonding With the Push-Pull Effect. Chem. Phys. Lett..

[129] Makoś M.Z., Freindorf M., Sethio D., Kraka E. (2019). New Insights into Fe−H_2_ and Fe−H^−^ Bonding of a [NiFe] Hydrogenase Mimic—A Local Vibrational Mode Study. Theor. Chem. Acc..

[130] Zhang X., Dai H., Yan H., Zou W., Cremer D. (2016). B-H *π* Interaction: A New Type of Nonclassical Hydrogen Bonding. J. Am. Chem. Soc..

[131] Zou W., Zhang X., Dai H., Yan H., Cremer D., Kraka E. (2018). Description of an Unusual Hydrogen Bond Between Carborane and a Phenyl Group. J. Organometal. Chem..

[132] Kalescky R., Kraka E., Cremer D. (2013). New Approach to Tolman’s Electronic Parameter Based on Local Vibrational Modes. Inorg. Chem..

[133] Setiawan D., Kalescky R., Kraka E., Cremer D. (2016). Direct Measure of Metal-Ligand Bonding Replacing the Tolman Electronic Parameter. Inorg. Chem..

[134] Li Y., Oliveira V., Tang C., Cremer D., Liu C., Ma J. (2017). The Peculiar Role of the Au_3_ Unit in Au_m_ Clusters: *σ*-Aromaticity of the Au_5_Zn^+^ Ion. Inorg. Chem..

[135] Cremer D., Kraka E. (2017). Generalization of the Tolman Electronic Parameter: The Metal-Ligand Electronic Parameter and the Intrinsic Strength of the Metal-Ligand Bond. Dalton Trans..

[136] Tao Y., Zou W., Sethio D., Verma N., Qiu Y., Tian C., Cremer D., Kraka E. (2019). In Situ Measure of Intrinsic Bond Strength in Crystalline Structures: Local Vibrational Mode Theory for Periodic Systems. J. Chem. Theory Comput..

[137] Frisch M.J., Trucks G.W., Schlegel H.B., Scuseria G.E., Robb M.A., Cheeseman J.R., Scalmani G., Barone V., Petersson G.A., Nakatsuji H. (2016). Gaussian16 Revision B.01.

[138] Neese F. (2011). The ORCA program system. Wiley Interdiscip. Rev. Comput. Mol. Sci..

[139] Neese F. (2018). Software update: The ORCA program system, version 4.0. Wiley Interdiscip. Rev. Comput. Mol. Sci..

[140] Lu T., Chen F. (2012). Multiwfn: A Multifunctional Wavefunction Analyzer. J. Comp. Chem..

[141] Kraka E., Zou W., Filatov M., Tao Y., Grafenstein J., Izotov D., Gauss J., He Y., Wu A., Konkoli Z. (2019). COLOGNE2019. http://www.smu.edu/catco.

[142] Bondi A. (1964). Van der waals volumes and radii. J. Phys. Chem..

[143] Rogachev A.Y., Hoffmann R. (2013). Hypervalent Compounds as Ligands: I_3_-Anion Adducts with Transition Metal Pentacarbonyls. Inorg. Chem..

