# Supplementary material for: Metal–Halogen Bonding Seen through the Eyes of Vibrational Spectroscopy
Source: Materials (Basel). 2019 Dec 20;13(1):55. doi: 10.3390/ma13010055 (PMC6982077; doi:10.3390/ma13010055)
Supplement: Supplementary file 1 [file materials-13-00055-s001.pdf]

Article

Supplementary Material
Metal halogen bonding - seen through the eyes of vibrational spectroscopy

Vytor P. Oliveira [1] 0000-0002-6614-7625, Bruna L. Marcial [2] 0000-0002-1014-7634, Francisco B. C. Machado [1] 0000-0002-2064-3463, and Elfi Kraka[3,*] 0000-0002-9658-5626

1. Affiliation 1; Instituto Tecnológico de Aeronáutica (ITA), Departamento de Química, São José dos Campos, 12228-900 São Paulo, Brazil; vytor3@gmail.com; fmachado@ita.br
2. Affiliation 2; Instituto Federal Goiano (IFGoiano), Núcleo de Química, Campus Morrinhos, Goias, Brazil; bruna.marcial@ifgoiano.edu.br
3. Affiliation 3; Department of Chemistry, Southern Methodist University, 3215 Daniel Avenue, Dallas, Texas 75275-0314, United States; ekraka@smu.edu;

* Correspondence: ekraka@smu.edu; Tel.: +1-214-768-2611

Version December 7, 2019 submitted to Materials

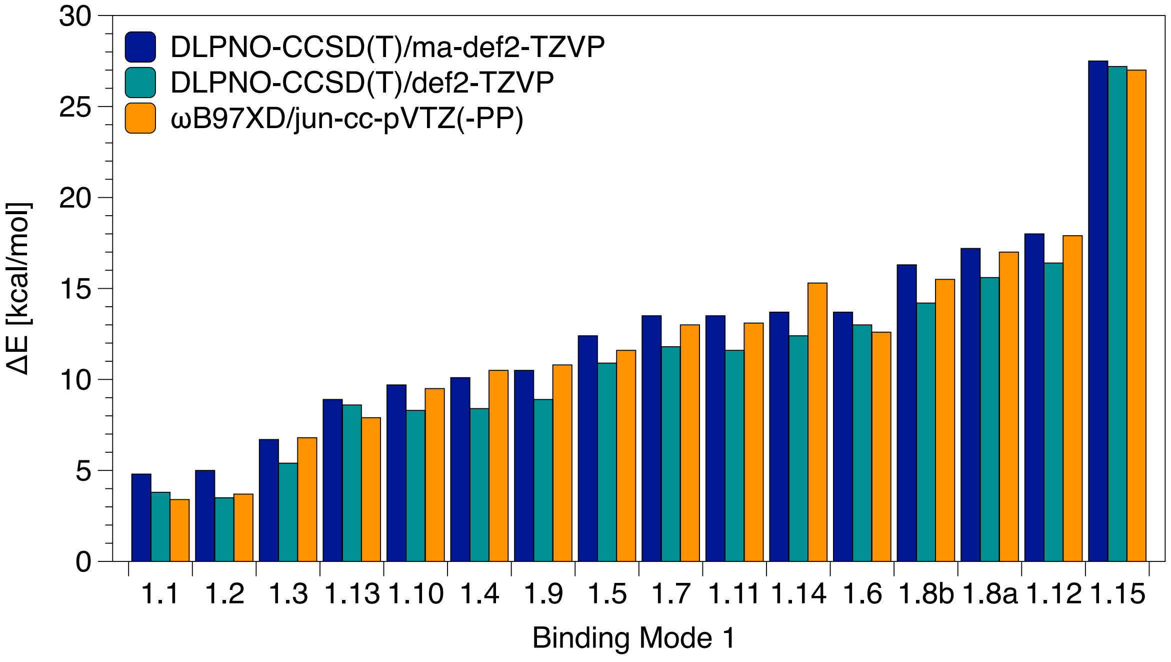

Figure S1. Ascendant ordering of binding energies obtained at different levels of theory for binding mode 1.

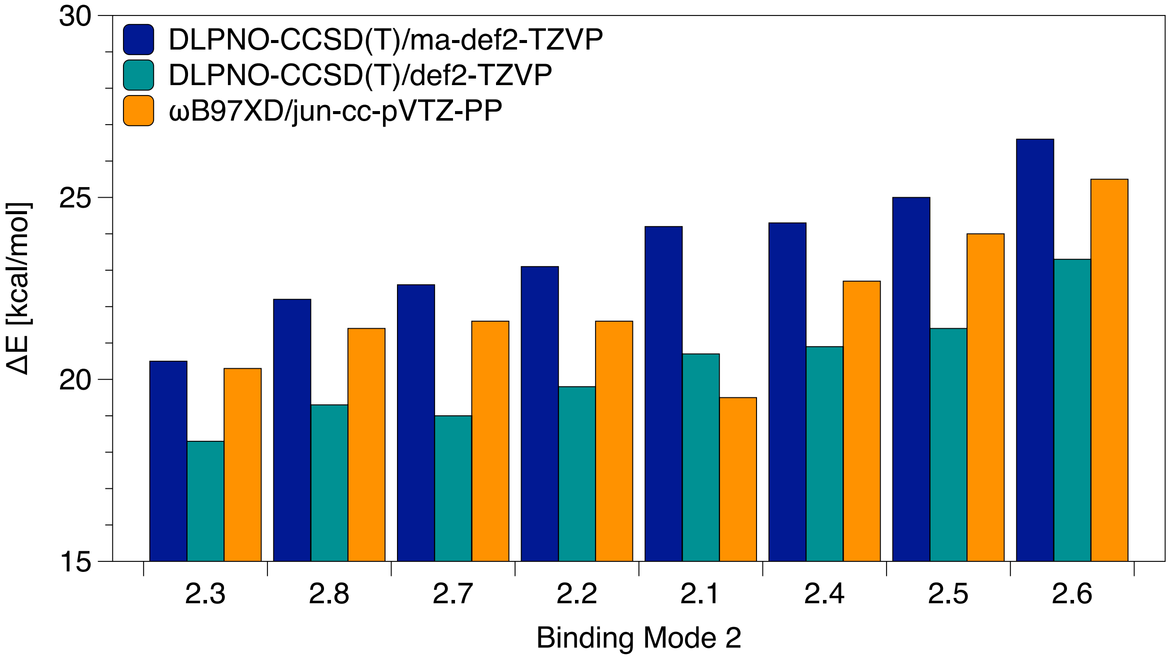

Figure S2. Ascendant ordering of binding energies obtained at different levels of theory for binding mode 2.

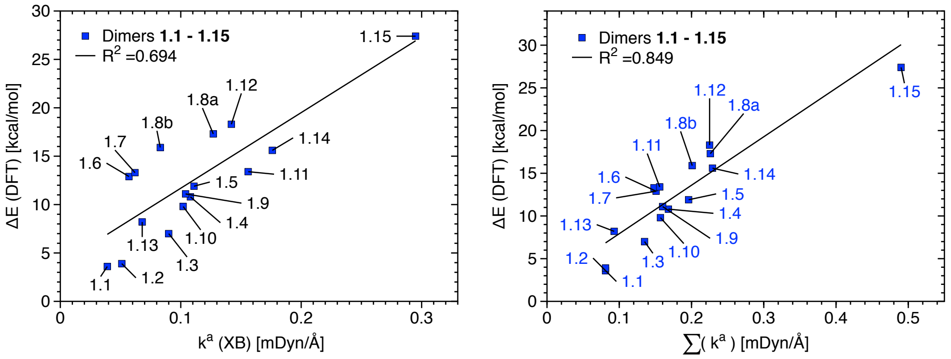

(a) (b)

Figure S3. Relationship between binding energies in kcal/mol and the XB local stretching force constant (k^a(XB)) in mdyn/Å(a), including others contributions beyond XB (b). Only binding mode 1 dimers (**1.1 - 1.15**) were considered.

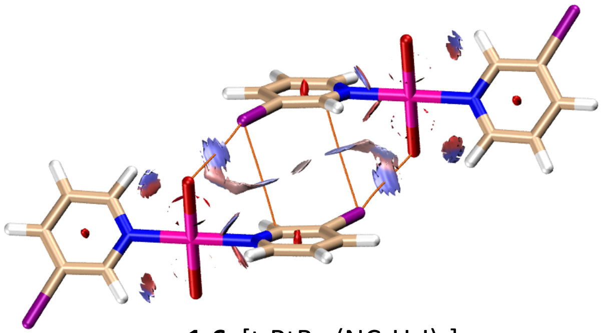

1.6 [t-$PtBr_2(NC_5H_4I)_2]_2$

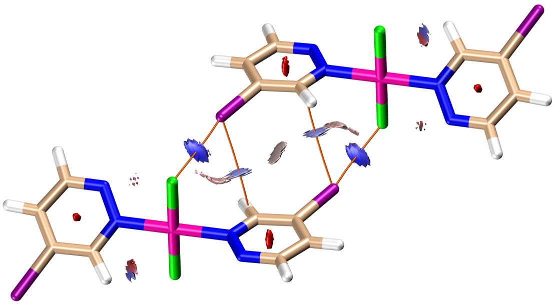

1.8a [t-$PtCl_2(N_2C_4H_3I)_2]_2$

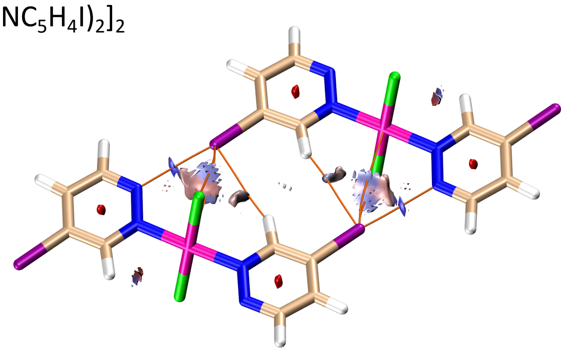

1.8b [t-$PtCl_2(N_2C_4H_3I)_2]_2$

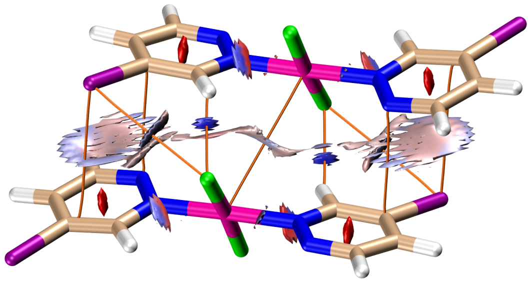

2.7 [t-$PtCl_2(N_2C_4H_3I)_2]_2$

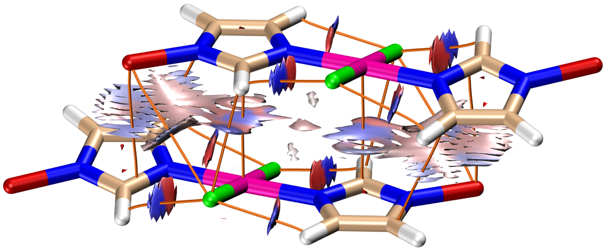

2.8 [t-$PtCl_2(N_2C_3H_3Br)_2]_2$

Figure S4. Colored RDG and bond paths. Dark blue regions indicate strong attractive interactions, light colors indicate dispersive interactions.

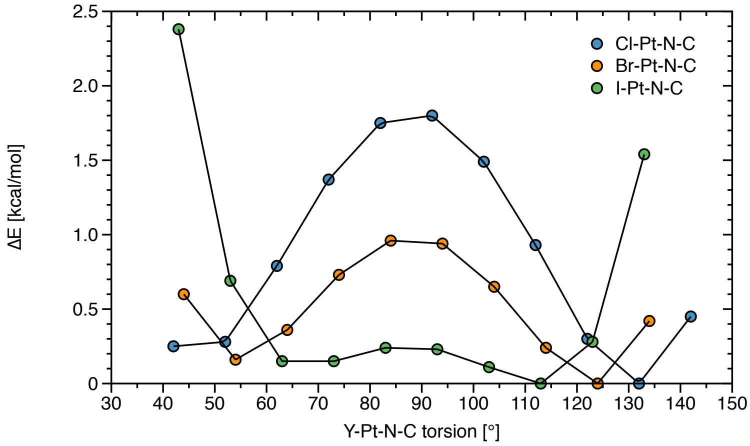

Figure S5. Relative energy increase associated with the torsion of the pyridine rings of dimers **1.4** (blue), **1.6** (orange), and **1.7** (green)

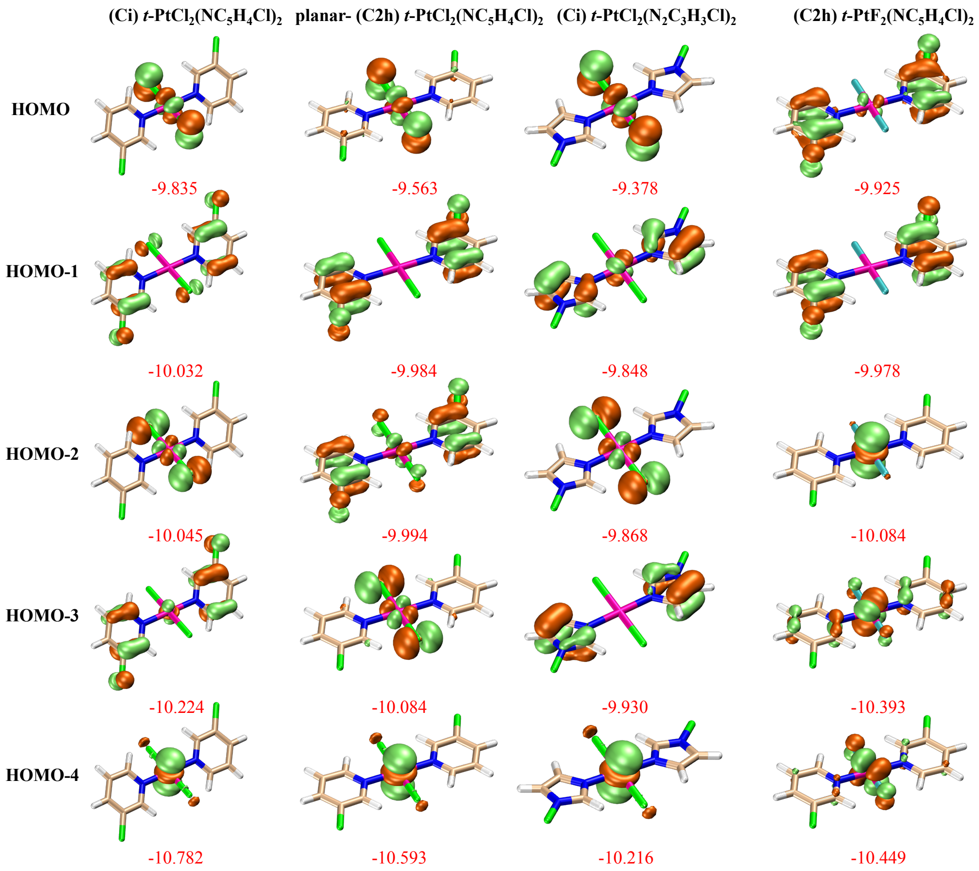

Figure S6. Selected molecular orbitals and orbital energies in eV. Calculated at HF/6-311g(d) with LANL2DZ ECP for Pt.

Table S1. Geometry, vibrational and topological properties of binding modes 2.

#	Dimer	Contact	Type	r	k[a]	BSO *n*	ρ_b	H_b
**2.1**	$[t{-}PdCl_2(NC_5H_4Cl)_2]_2$	$H\cdots Cl$	HB	2.818	0.038	0.113	0.008	0.167
**2.1**	$[t{-}PdCl_2(NC_5H_4Cl)_2]_2$	$H\cdots Cl$	HB	2.800	0.048	0.128	0.008	0.172
**2.1**	$[t{-}PdCl_2(NC_5H_4Cl)_2]_2$	$Pd\cdots[Pd\text{-}Cl]$	NS	3.525	0.170	0.259	0.045	0.003
**2.1**	$[t{-}PdCl_2(NC_5H_4Cl)_2]_2$	$[C{-}Cl]\cdots[C_5N]$	NS	3.539	0.105	0.198		
**2.2**	$[t{-}PtCl_2(NC_5H_4Cl)_2]_2$	$H\cdots Cl$	HB	2.886	0.040	0.116	0.007	0.143
**2.2**	$[t{-}PtCl_2(NC_5H_4Cl)_2]_2$	$H\cdots Cl$	HB	2.842	0.036	0.109	0.008	0.156
**2.2**	$[t{-}PtCl_2(NC_5H_4Cl)_2]_2$	$Pt\cdots[Pt\text{-}Cl]$	NS	3.635	0.186	0.272	0.055	0.003
**2.2**	$[t{-}PtCl_2(NC_5H_4Cl)_2]_2$	$[C{-}Cl]\cdots[C_5N]$	NS	3.513	0.133	0.226		
**2.3**	$[t{-}PtCl_2(NC_5H_4F)_2]_2$	$H\cdots F$	HB	2.865	0.044	0.122	0.008	0.151
**2.3**	$[t{-}PtCl_2(NC_5H_4F)_2]_2$	$H\cdots F$	HB	2.874	0.046	0.125	0.007	0.146
**2.3**	$[t{-}PtCl_2(NC_5H_4F)_2]_2$	$Pt\cdots[Pt\text{-}Cl]$	NS	3.638	0.195	0.279	0.055	0.003
**2.3**	$[t{-}PtCl_2(NC_5H_4F)_2]_2$	$[C{-}Br]\cdots[C_5N]$	NS	3.359	0.100	0.193		
**2.4**	$[t{-}PtCl_2(NC_5H_4Br)_2]_2$	$H\cdots Cl$	HB	2.854	0.036	0.109	0.007	0.151
**2.4**	$[t{-}PtCl_2(NC_5H_4Br)_2]_2$	$H\cdots Cl$	HB	2.872	0.043	0.121	0.007	0.147
**2.4**	$[t{-}PtCl_2(NC_5H_4Br)_2]_2$	$Pt\cdots[Pt\text{-}Cl]$	NS	3.639	0.186	0.272	0.055	0.003
**2.4**	$[t{-}PtCl_2(NC_5H_4Br)_2]_2$	$[C{-}Br]\cdots[C_5N]$	NS	3.581	0.149	0.241		
**2.5**	$[t{-}PtCl_2(NC_5H_4I)_2]_2$	$H\cdots Cl$	HB	2.882	0.035	0.108	0.007	0.142
**2.5**	$[t{-}PtCl_2(NC_5H_4I)_2]_2$	$H\cdots Cl$	HB	2.846	0.037	0.111	0.008	0.156
**2.5**	$[t{-}PtCl_2(NC_5H_4I)_2]_2$	$Pt\cdots[Pt{-}Cl]$	NS	3.650	0.187	0.273	0.054	0.003
**2.5**	$[t{-}PtCl_2(NC_5H_4I)_2]_2$	$[C{-}I]\cdots[C_5N]$	NS	3.705	0.124	0.217		
**2.6**	$[t{-}PtBr_2(NC_5H_4I)_2]_2$	$H\cdots Br$	HB	2.888	0.052	0.134	0.006	0.155
**2.6**	$[t{-}PtBr_2(NC_5H_4I)_2]_2$	$H\cdots Br$	HB	2.879	0.063	0.149	0.006	0.161
**2.6**	$[t{-}PtBr_2(NC_5H_4I)_2]_2$	$Pt\cdots[Pt{-}Br]$	NS	3.780	0.169	0.258	0.043	0.003
**2.6**	$[t{-}PtBr_2(NC_5H_4I)_2]_2$	$[C{-}I]\cdots[C_5N]$	NS	3.696	0.138	0.231		
**2.7**	$[t{-}PtCl_2(N_2C_4H_3I)_2]_2$	$Cl\cdots[C\text{-}H]$	NS	2.972	0.140	0.232		
**2.7**	$[t{-}PtCl_2(N_2C_4H_3I)_2]_2$	$[C{-}I]\cdots[C_3N_2]$	NS	3.549	0.138	0.231		
**2.8**	$[t{-}PtCl_2(N_2C_3H_3Br)_2]_2$	$Cl\cdots H$	HB	2.983	0.056	0.140		
**2.8**	$[t{-}PtCl_2(N_2C_3H_3Br)_2]_2$	$[N{-}Br]\cdots[C_3N_2]$	NS	3.503	0.093	0.185		
**2.8**	$[t{-}PtCl_2(N_2C_3H_3Br)_2]_2$	$Pt\cdots[C_3N_2]$	NS	3.510	0.139	0.231		

[a]Atom-atom specific interaction are hydrogen bond HB; non-specific (NS) interaction involve the mid-point of a bond or a ring-plane (in brackets), r is the distance in Å involving atoms, mid-points or ring-planes, r(vdW) are the sum of Bondi radii of the closest two atoms. Local stretching force constant (k[a]) in mdyn/Å, density at the BCP (ρ_b) in e/Å^3 and energy density at the BCP in Hartree/Å^3.

Table S2. GEOMETRIES AT ωB97XD/jun-cc-pVTZ(-PP)

Dimers: **1.1** $[t{-}PdCl_2(NC_5H_4Cl)_2]_2$					Monomer: $t{-}PdCl_2(NC_5H_4Cl)_2$			
Index	Atom type	*x*	*y*	*z*	Atom type	*x*	*y*	*z*
1	C	0.643	3.125	-3.682	C	-0.654	-1.736	2.297
2	C	0.809	4.059	-2.680	C	-0.800	-2.119	3.614
3	C	0.491	3.722	-1.378	C	-0.442	-1.243	4.622
4	C	0.021	2.445	-1.130	C	0.045	-0.002	4.258
5	C	-0.105	1.547	-2.172	C	0.152	0.334	2.922
6	H	0.847	3.354	-4.716	H	-0.890	-2.400	1.480
7	H	1.177	5.043	-2.926	H	-1.185	-3.101	3.843
8	H	0.599	4.430	-0.569	H	-0.536	-1.512	5.664
9	H	-0.442	0.536	-2.005	H	0.496	1.308	2.610
10	N	0.199	1.891	-3.425	N	-0.189	-0.528	1.963
11	Pd	-0.018	0.546	-4.938	Pd	0.000	0.000	0.000
12	Cl	1.191	-1.011	-3.723	Cl	-1.002	2.035	0.453
13	Cl	-1.203	2.115	-6.151	Cl	1.002	-2.035	-0.453
14	Cl	-0.413	1.959	0.471	C	-0.152	-0.334	-2.922
15	C	-0.032	-0.428	-7.721	C	0.654	1.736	-2.297
16	C	-0.595	-2.064	-6.194	C	-0.045	0.002	-4.258
17	C	-0.166	-1.323	-8.765	H	-0.496	-1.308	-2.610
18	H	0.226	0.606	-7.891	C	0.800	2.119	-3.614
19	C	-0.762	-2.997	-7.197	H	0.890	2.400	-1.480
20	H	-0.724	-2.316	-5.153	C	0.442	1.243	-4.622
21	C	-0.541	-2.629	-8.511	H	1.185	3.101	-3.843
22	H	-1.056	-4.004	-6.945	H	0.536	1.512	-5.664
23	H	-0.656	-3.334	-9.322	N	0.189	0.528	-1.963
24	N	-0.240	-0.803	-6.457	Cl	0.520	1.151	5.458
25	Cl	0.135	-0.785	-10.381	Cl	-0.520	-1.151	-5.458
26	C	-0.021	-2.445	1.130				
27	C	0.105	-1.547	2.172				
28	C	-0.643	-3.125	3.682				
29	C	-0.809	-4.059	2.680				
30	C	-0.491	-3.722	1.378				
31	H	0.442	-0.536	2.005				
32	H	-0.847	-3.354	4.716				
33	H	-1.177	-5.043	2.926				
34	H	-0.599	-4.430	0.569				
35	Pd	0.018	-0.546	4.938				
36	Cl	-1.191	1.011	3.723				
37	Cl	1.203	-2.115	6.151				
38	Cl	0.413	-1.959	-0.471				
39	N	-0.199	-1.891	3.425				
40	C	0.032	0.428	7.721				
41	C	0.595	2.064	6.194				
42	C	0.166	1.323	8.765				
43	H	-0.226	-0.606	7.891				
44	C	0.762	2.997	7.197				
45	H	0.724	2.316	5.153				

46	C	0.541	2.629	8.511
47	H	1.056	4.004	6.945
48	H	0.656	3.334	9.322
49	N	0.240	0.803	6.457
50	Cl	-0.135	0.785	10.381

Dimers: **1.2** [t−$PtCl_2(NC_5H_4Cl)_2]_2$ Monomer: t−$PtCl_2(NC_5H_4Cl)_2$

Index	Atom type	*x*	*y*	*z*	Atom type	*x*	*y*	*z*
1	C	0.648	3.154	-3.652	C	-0.657	-1.734	2.288
2	C	0.813	4.096	-2.659	C	-0.800	-2.116	3.606
3	C	0.519	3.764	-1.349	C	-0.442	-1.241	4.614
4	C	0.072	2.482	-1.087	C	0.047	-0.001	4.249
5	C	-0.059	1.575	-2.121	C	0.157	0.335	2.915
6	H	0.839	3.378	-4.690	H	-0.901	-2.395	1.472
7	H	1.163	5.083	-2.917	H	-1.187	-3.097	3.834
8	H	0.629	4.479	-0.547	H	-0.536	-1.509	5.656
9	H	-0.386	0.563	-1.943	H	0.509	1.306	2.603
10	N	0.223	1.914	-3.383	N	-0.188	-0.526	1.952
11	Pt	-0.001	0.562	-4.877	Pt	0.000	0.000	0.000
12	Cl	1.273	-0.967	-3.685	Cl	-1.032	2.027	0.448
13	Cl	-1.255	2.100	-6.070	Cl	1.032	-2.027	-0.448
14	Cl	-0.336	1.998	0.521	C	-0.157	-0.335	-2.915
15	C	-0.036	-0.425	-7.646	C	0.657	1.734	-2.288
16	C	-0.576	-2.056	-6.101	C	-0.047	0.001	-4.249
17	C	-0.179	-1.327	-8.681	H	-0.509	-1.306	-2.603
18	H	0.219	0.608	-7.825	C	0.800	2.116	-3.606
19	C	-0.745	-2.994	-7.098	H	0.901	2.395	-1.472
20	H	-0.698	-2.300	-5.058	C	0.442	1.241	-4.614
21	C	-0.542	-2.634	-8.416	H	1.187	3.097	-3.834
22	H	-1.029	-4.001	-6.835	H	0.536	1.509	-5.656
23	H	-0.660	-3.344	-9.222	N	0.188	0.526	-1.952
24	N	-0.230	-0.791	-6.374	Cl	0.523	1.152	5.448
25	Cl	0.100	-0.795	-10.304	Cl	-0.523	-1.152	-5.448
26	N	0.230	0.791	6.374				
27	C	-0.072	-2.482	1.087				
28	C	0.059	-1.575	2.121				
29	C	-0.648	-3.154	3.652				
30	C	-0.813	-4.096	2.659				
31	C	-0.519	-3.764	1.349				
32	H	0.386	-0.563	1.943				
33	H	-0.839	-3.378	4.690				
34	H	-1.163	-5.083	2.917				
35	H	-0.629	-4.479	0.547				
36	Pt	0.001	-0.562	4.877				
37	Cl	-1.273	0.967	3.685				
38	Cl	1.255	-2.100	6.070				
39	Cl	0.336	-1.998	-0.521				
40	N	-0.223	-1.914	3.383				
41	C	0.036	0.425	7.646				

42	C	0.576	2.056	6.101
43	C	0.179	1.327	8.681
44	H	-0.219	-0.608	7.825
45	C	0.745	2.994	7.098
46	H	0.698	2.300	5.058
47	C	0.542	2.634	8.416
48	H	1.029	4.001	6.835
49	H	0.660	3.344	9.222
50	Cl	-0.100	0.795	10.304

Dimers: **1.3** [t−$PtCl_2(NC_5H_4Br)_2]_2$

Index	Atom type	*x*	*y*	*z*
1	C	0.035	-1.684	2.049
2	C	-0.702	-3.278	3.549
3	C	-0.852	-4.206	2.540
4	C	-0.535	-3.857	1.240
5	H	0.371	-0.671	1.895
6	H	-0.911	-3.515	4.580
7	H	-1.208	-5.196	2.780
8	H	-0.635	-4.566	0.431
9	Pt	-0.066	-0.700	4.812
10	N	-0.271	-2.035	3.303
11	C	-0.007	0.249	7.595
12	C	0.455	1.915	6.064
13	H	-0.229	-0.794	7.760
14	C	0.616	2.845	7.070
15	H	0.552	2.178	5.023
16	C	0.449	2.460	8.387
17	H	0.867	3.863	6.816
18	H	0.563	3.167	9.196
19	N	0.151	0.638	6.325
20	Br	0.406	-2.015	-0.734
21	C	-0.079	-2.574	0.999
22	C	0.129	1.140	8.641
23	Cl	-1.420	0.805	3.674
24	Cl	1.250	-2.221	5.956
25	Br	-0.123	0.526	10.405
26	C	0.702	3.278	-3.549
27	C	0.852	4.206	-2.540
28	C	0.535	3.857	-1.240
29	C	-0.035	1.684	-2.049
30	H	0.911	3.515	-4.580
31	H	1.208	5.196	-2.780
32	H	0.635	4.566	-0.431
33	H	-0.371	0.671	-1.895
34	N	0.271	2.035	-3.303
35	Pt	0.066	0.700	-4.812
36	C	0.007	-0.249	-7.595
37	C	-0.455	-1.915	-6.064

Monomer: t−$PtCl_2(NC_5H_4Br)_2$

Atom type	*x*	*y*	*z*
C	0.012	0.066	-2.938
C	-0.629	-1.983	-2.085
C	-0.800	-2.497	-3.353
C	-0.545	-1.700	-4.453
H	0.308	1.082	-2.728
H	-0.791	-2.578	-1.201
H	-1.126	-3.519	-3.471
H	-0.665	-2.076	-5.459
Pt	0.000	0.000	0.000
N	-0.232	-0.721	-1.883
C	-0.012	-0.066	2.938
C	0.629	1.983	2.085
H	-0.308	-1.082	2.728
C	0.800	2.497	3.353
H	0.791	2.578	1.201
C	0.545	1.700	4.453
H	1.126	3.519	3.471
H	0.665	2.076	5.459
N	0.232	0.721	1.883
Br	0.245	0.758	-5.667
Br	-0.245	-0.758	5.667
C	-0.130	-0.401	-4.229
C	0.130	0.401	4.229
Cl	-1.186	1.905	-0.585
Cl	1.186	-1.905	0.585

38	H	0.229	0.794	-7.760
39	C	-0.616	-2.845	-7.070
40	H	-0.552	-2.178	-5.023
41	C	-0.449	-2.460	-8.387
42	H	-0.867	-3.863	-6.816
43	H	-0.563	-3.167	-9.196
44	N	-0.151	-0.638	-6.325
45	Br	-0.406	2.015	0.734
46	Br	0.123	-0.526	-10.405
47	C	0.079	2.574	-0.999
48	C	-0.129	-1.140	-8.641
49	Cl	1.420	-0.805	-3.674
50	Cl	-1.250	2.221	-5.956

Dimers: **1.4** [t−$PtCl_2(NC_5H_4I)_2]_2$ Monomer: t−$PtCl_2(NC_5H_4I)_2$

Index	Atom type	*x*	*y*	*z*	Atom type	*x*	*y*	*z*
1	C	0.815	3.389	-3.545	C	-0.033	-0.095	-2.937
2	C	0.996	4.296	-2.522	C	-0.571	-2.120	-1.963
3	C	0.655	3.942	-1.229	C	-0.739	-2.709	-3.198
4	C	-0.003	1.811	-2.070	C	-0.536	-1.961	-4.344
5	H	1.043	3.629	-4.571	H	0.221	0.941	-2.780
6	H	1.396	5.273	-2.745	H	-0.693	-2.672	-1.045
7	H	0.783	4.641	-0.415	H	-1.022	-3.749	-3.256
8	H	-0.388	0.812	-1.941	H	-0.656	-2.404	-5.322
9	N	0.330	2.163	-3.317	Pt	0.000	0.000	0.000
10	Pt	0.072	0.854	-4.839	N	-0.227	-0.833	-1.837
11	C	-0.093	-0.051	-7.634	C	0.033	0.095	2.937
12	C	-0.512	-1.738	-6.112	C	0.571	2.120	1.963
13	H	0.131	0.993	-7.782	H	-0.221	-0.941	2.780
14	C	-0.718	-2.649	-7.126	C	0.739	2.709	3.198
15	H	-0.575	-2.017	-5.072	H	0.693	2.672	1.045
16	C	-0.596	-2.244	-8.442	C	0.536	1.961	4.344
17	H	-0.967	-3.670	-6.879	H	1.022	3.749	3.256
18	H	-0.747	-2.942	-9.252	H	0.656	2.404	5.322
19	N	-0.208	-0.459	-6.365	N	0.227	0.833	1.837
20	I	-0.443	2.037	0.907	I	0.161	0.581	-5.870
21	I	-0.063	-0.210	-10.646	I	-0.161	-0.581	5.870
22	C	0.140	2.676	-1.000	C	-0.173	-0.633	-4.203
23	C	-0.274	-0.921	-8.693	C	0.173	0.633	4.203
24	Cl	1.479	-0.671	-3.788	Cl	-1.274	1.818	-0.668
25	Cl	-1.278	2.399	-5.904	Cl	1.274	-1.818	0.668
26	N	0.208	0.459	6.365				
27	I	0.443	-2.037	-0.907				
28	C	0.003	-1.811	2.070				
29	C	-0.815	-3.389	3.545				
30	C	-0.996	-4.296	2.522				
31	C	-0.655	-3.942	1.229				
32	H	0.388	-0.812	1.941				
33	H	-1.043	-3.629	4.571				

34	H	-1.396	-5.273	2.745
35	H	-0.783	-4.641	0.415
36	Pt	-0.072	-0.854	4.839
37	N	-0.330	-2.163	3.317
38	C	0.093	0.051	7.634
39	C	0.512	1.738	6.112
40	H	-0.131	-0.993	7.782
41	C	0.718	2.649	7.126
42	H	0.575	2.017	5.072
43	C	0.596	2.244	8.442
44	H	0.967	3.670	6.879
45	H	0.747	2.942	9.252
46	I	0.063	0.210	10.646
47	C	-0.140	-2.676	1.000
48	C	0.274	0.921	8.693
49	Cl	-1.479	0.671	3.788
50	Cl	1.278	-2.399	5.904

Dimers: **1.5** $[t{-}PtF_2(NC_5H_4I)_2]_2$ Monomer: $t{-}PtF_2(NC_5H_4I)_2$

Index	Atom type	*x*	*y*	*z*	Atom type	*x*	*y*	*z*
1	C	-2.406	3.938	1.468	C	2.700	1.156	0.000
2	C	-3.368	3.078	1.952	C	4.079	1.184	0.000
3	C	-3.336	1.741	1.592	C	4.786	-0.005	0.000
4	C	-2.339	1.308	0.734	C	4.074	-1.193	0.000
5	C	-1.416	2.226	0.266	C	2.692	-1.161	0.000
6	H	-2.373	4.989	1.714	H	2.087	2.046	0.000
7	H	-4.131	3.456	2.616	H	4.592	2.134	0.000
8	H	-4.072	1.049	1.977	H	5.867	-0.006	0.000
9	H	-0.617	1.953	-0.405	H	2.074	-2.048	0.000
10	N	-1.442	3.511	0.641	N	2.025	0.000	0.000
11	Pt	0.003	4.786	0.013	Pt	0.000	0.000	0.000
12	I	-2.121	-0.698	0.176	I	5.054	-3.039	0.000
13	C	1.316	7.385	-0.331	C	-2.692	1.161	0.000
14	C	2.514	5.639	-1.270	C	-2.700	-1.156	0.000
15	C	2.276	8.295	-0.733	C	-4.074	1.193	0.000
16	H	0.420	7.662	0.207	H	-2.074	2.048	0.000
17	C	3.501	6.502	-1.696	C	-4.079	-1.184	0.000
18	H	2.543	4.573	-1.445	H	-2.087	-2.046	0.000
19	C	3.390	7.854	-1.427	C	-4.786	0.005	0.000
20	H	4.351	6.112	-2.235	H	-4.592	-2.134	0.000
21	H	4.153	8.547	-1.751	H	-5.867	0.006	0.000
22	N	1.442	6.079	-0.599	N	-2.025	0.000	0.000
23	I	2.019	10.319	-0.280	I	-5.054	3.039	0.000
24	F	-1.066	6.300	0.682	F	0.000	1.978	0.000
25	F	1.100	3.270	-0.665	F	0.000	-1.978	0.000
26	N	-1.442	-6.079	-0.599				
27	C	2.339	-1.308	0.734				
28	C	1.416	-2.226	0.266				
29	C	2.406	-3.938	1.468				

30	C	3.368	-3.078	1.952
31	C	3.336	-1.741	1.592
32	H	0.617	-1.953	-0.405
33	H	2.373	-4.989	1.714
34	H	4.131	-3.456	2.616
35	H	4.072	-1.049	1.977
36	Pt	-0.003	-4.786	0.013
37	I	2.121	0.698	0.176
38	N	1.442	-3.511	0.641
39	C	-1.316	-7.385	-0.331
40	C	-2.514	-5.639	-1.270
41	C	-2.276	-8.295	-0.733
42	H	-0.420	-7.662	0.207
43	C	-3.501	-6.502	-1.696
44	H	-2.543	-4.573	-1.445
45	C	-3.390	-7.854	-1.427
46	H	-4.351	-6.112	-2.235
47	H	-4.153	-8.547	-1.751
48	I	-2.019	-10.319	-0.280
49	F	-1.100	-3.270	-0.665
50	F	1.066	-6.300	0.682

Dimers: **1.6** [t−$PtBr_2(NC_5H_4I)_2]_2$ Monomer: t−$PtBr_2(NC_5H_4I)_2$

Index	Atom type	*x*	*y*	*z*	Atom type	*x*	*y*	*z*
1	C	1.337	2.697	-2.797	C	1.081	1.915	1.958
2	C	1.566	3.484	-1.689	C	1.368	2.460	3.192
3	C	0.823	3.275	-0.541	C	0.925	1.825	4.337
4	C	-0.346	1.554	-1.705	C	-0.039	0.147	2.934
5	H	1.917	2.801	-3.701	H	1.388	2.390	1.040
6	H	2.333	4.242	-1.726	H	1.932	3.379	3.248
7	H	1.003	3.866	0.346	H	1.134	2.236	5.314
8	H	-1.104	0.789	-1.770	H	-0.574	-0.776	2.781
9	N	0.394	1.749	-2.803	N	0.391	0.776	1.834
10	Pt	0.121	0.559	-4.422	Pt	0.000	0.000	0.000
11	C	-0.575	-0.136	-7.192	C	0.039	-0.147	-2.934
12	C	0.068	-1.965	-5.938	C	-1.081	-1.915	-1.958
13	H	-0.760	0.927	-7.208	H	0.574	0.776	-2.781
14	C	-0.124	-2.811	-7.009	C	-1.368	-2.460	-3.192
15	H	0.416	-2.324	-4.982	H	-1.388	-2.390	-1.040
16	C	-0.548	-2.293	-8.219	C	-0.925	-1.825	-4.337
17	H	0.065	-3.867	-6.891	H	-1.932	-3.379	-3.248
18	H	-0.700	-2.937	-9.073	H	-1.134	-2.236	-5.314
19	N	-0.157	-0.649	-6.030	N	-0.391	-0.776	-1.834
20	I	-1.251	1.807	1.161	I	-0.512	-0.385	5.864
21	I	-1.415	-0.049	-10.087	I	0.512	0.385	-5.864
22	C	-0.146	2.287	-0.550	C	0.206	0.650	4.198
23	C	-0.773	-0.930	-8.306	C	-0.206	-0.650	-4.198
24	Br	2.448	-0.229	-4.232	Br	1.040	-2.117	0.675
25	Br	-2.192	1.330	-4.597	Br	-1.040	2.117	-0.675

26	N	0.157	0.649	6.030
27	I	1.251	-1.807	-1.161
28	C	0.346	-1.554	1.705
29	C	-1.337	-2.697	2.797
30	C	-1.566	-3.484	1.689
31	C	-0.823	-3.275	0.541
32	H	1.104	-0.789	1.770
33	H	-1.917	-2.801	3.701
34	H	-2.333	-4.242	1.726
35	H	-1.003	-3.866	-0.346
36	Pt	-0.121	-0.559	4.422
37	N	-0.394	-1.749	2.803
38	C	0.575	0.136	7.192
39	C	-0.068	1.965	5.938
40	H	0.760	-0.927	7.208
41	C	0.124	2.811	7.009
42	H	-0.416	2.324	4.982
43	C	0.548	2.293	8.219
44	H	-0.065	3.867	6.891
45	H	0.700	2.937	9.073
46	I	1.415	0.049	10.087
47	C	0.146	-2.287	0.550
48	C	0.773	0.930	8.306
49	Br	-2.448	0.229	4.232
50	Br	2.192	-1.330	4.597

Dimers: **1.7** $[t{-}PtI_2(NC_5H_4I)_2]_2$ Monomer: $t{-}PtI_2(NC_5H_4I)_2$

Index	Atom type	*x*	*y*	*z*	Atom type	*x*	*y*	*z*
1	C	1.176	2.907	-2.958	C	-0.671	-2.605	1.200
2	C	1.377	3.733	-1.874	C	-0.885	-3.542	2.188
3	C	0.720	3.469	-0.685	C	-0.689	-3.193	3.512
4	C	-0.314	1.611	-1.763	C	-0.102	-1.002	2.760
5	H	1.689	3.060	-3.894	H	-0.795	-2.841	0.155
6	H	2.055	4.568	-1.962	H	-1.197	-4.539	1.916
7	H	0.877	4.095	0.182	H	-0.846	-3.912	4.302
8	H	-0.988	0.770	-1.775	H	0.192	0.020	2.937
9	N	0.342	1.862	-2.901	N	-0.289	-1.355	1.484
10	Pt	0.089	0.633	-4.497	Pt	0.000	0.000	0.000
11	C	-0.320	-0.118	-7.311	C	0.102	1.002	-2.760
12	C	-0.211	-1.938	-5.895	C	0.671	2.605	-1.200
13	H	-0.299	0.956	-7.402	H	-0.192	-0.020	-2.937
14	C	-0.399	-2.811	-6.945	C	0.885	3.542	-2.188
15	H	-0.079	-2.285	-4.882	H	0.795	2.841	-0.155
16	C	-0.544	-2.313	-8.227	C	0.689	3.193	-3.512
17	H	-0.425	-3.873	-6.755	H	1.197	4.539	-1.916
18	H	-0.688	-2.979	-9.066	H	0.846	3.912	-4.302
19	N	-0.175	-0.614	-6.078	N	0.289	1.355	-1.484
20	I	-1.140	1.840	1.126	I	0.035	-1.269	5.760
21	I	-0.711	-0.087	-10.300	I	-0.035	1.269	-5.760

22	C	-0.138	2.384	-0.630	C	-0.287	-1.899	3.795
23	C	-0.500	-0.941	-8.407	C	0.287	1.899	-3.795
24	I	2.585	-0.221	-4.260	I	-2.065	1.393	0.873
25	I	-2.393	1.469	-4.754	I	2.065	-1.393	-0.873
26	N	0.175	0.614	6.078				
27	I	1.140	-1.840	-1.126				
28	C	0.314	-1.611	1.763				
29	C	-1.176	-2.907	2.958				
30	C	-1.377	-3.733	1.874				
31	C	-0.720	-3.469	0.685				
32	H	0.988	-0.770	1.775				
33	H	-1.689	-3.060	3.894				
34	H	-2.055	-4.568	1.962				
35	H	-0.877	-4.095	-0.182				
36	Pt	-0.089	-0.633	4.497				
37	N	-0.342	-1.862	2.901				
38	C	0.320	0.118	7.311				
39	C	0.211	1.938	5.895				
40	H	0.299	-0.956	7.402				
41	C	0.399	2.811	6.945				
42	H	0.079	2.285	4.882				
43	C	0.544	2.313	8.227				
44	H	0.425	3.873	6.755				
45	H	0.688	2.979	9.066				
46	I	0.711	0.087	10.300				
47	C	0.138	-2.384	0.630				
48	C	0.500	0.941	8.407				
49	I	-2.585	0.221	4.260				
50	I	2.393	-1.469	4.754				

Dimers: **1.8a** $[t{-}PtCl_2(N_2C_4H_3I)_2]_2$ Monomer: $t{-}PtCl_2(N_2C_4H_3I)_2$

Index	Atom type	*x*	*y*	*z*	Atom type	*x*	*y*	*z*
1	N	1.190	2.863	-2.993	N	1.883	2.100	1.065
2	C	1.387	3.635	-1.942	C	2.349	3.334	1.065
3	C	0.775	3.439	-0.709	C	1.675	4.418	0.515
4	C	-0.317	1.627	-1.745	C	0.000	2.850	-0.055
5	H	2.087	4.443	-2.099	H	3.316	3.454	1.533
6	H	1.002	4.085	0.126	H	2.102	5.409	0.549
7	H	-1.005	0.797	-1.770	H	-0.934	2.550	-0.503
8	N	0.342	1.865	-2.872	N	0.712	1.879	0.511
9	Pt	0.106	0.634	-4.445	Pt	0.000	0.000	0.522
10	C	-0.315	-0.095	-7.232	C	0.000	-2.850	-0.055
11	N	-0.153	-1.895	-5.784	N	-1.883	-2.100	1.065
12	H	-0.322	0.982	-7.298	H	0.934	-2.550	-0.503
13	C	-0.318	-2.708	-6.809	C	-2.349	-3.334	1.065
14	C	-0.484	-2.279	-8.120	C	-1.675	-4.418	0.515
15	H	-0.316	-3.759	-6.559	H	-3.316	-3.454	1.533
16	H	-0.610	-2.991	-8.922	H	-2.102	-5.409	0.549
17	N	-0.148	-0.600	-6.012	N	-0.712	-1.879	0.511

18	I	-1.035	1.840	1.188	I	-0.694	5.653	-0.952
19	I	-0.727	-0.090	-10.227	I	0.694	-5.653	-0.952
20	C	-0.108	2.390	-0.601	C	0.457	4.160	-0.067
21	C	-0.483	-0.922	-8.334	C	-0.457	-4.160	-0.067
22	Cl	2.137	-0.384	-3.941	Cl	0.000	0.000	2.831
23	Cl	-1.911	1.655	-4.939	Cl	0.000	0.000	-1.805
24	N	0.148	0.600	6.012				
25	I	1.035	-1.840	-1.188				
26	C	0.317	-1.627	1.745				
27	N	-1.190	-2.863	2.993				
28	C	-1.387	-3.635	1.942				
29	C	-0.775	-3.439	0.709				
30	H	1.005	-0.797	1.770				
31	H	-2.087	-4.443	2.099				
32	H	-1.002	-4.085	-0.126				
33	Pt	-0.106	-0.634	4.445				
34	N	-0.342	-1.865	2.872				
35	C	0.315	0.095	7.232				
36	N	0.153	1.895	5.784				
37	H	0.322	-0.982	7.298				
38	C	0.318	2.708	6.809				
39	C	0.484	2.279	8.120				
40	H	0.316	3.759	6.559				
41	H	0.610	2.991	8.922				
42	I	0.727	0.090	10.227				
43	C	0.108	-2.390	0.601				
44	C	0.483	0.922	8.334				
45	Cl	-2.137	0.384	3.941				
46	Cl	1.911	-1.655	4.939				

Dimers: **1.8b** $[t\text{−}PtCl_2(N_2C_4H_3I)_2]_2$

Index	Atom type	*x*	*y*	*z*
1	N	2.759	3.805	0.302
2	C	1.784	4.695	0.292
3	C	0.436	4.370	0.198
4	C	1.167	2.131	0.154
5	H	2.110	5.723	0.358
6	H	-0.316	5.145	0.182
7	H	1.020	1.063	0.117
8	N	2.430	2.535	0.230
9	Pt	3.892	1.160	0.216
10	C	6.594	0.081	0.037
11	N	4.931	-1.511	0.262
12	H	6.793	1.137	-0.059
13	C	5.851	-2.454	0.229
14	C	7.212	-2.201	0.110
15	H	5.471	-3.464	0.302
16	H	7.925	-3.012	0.094
17	N	5.315	-0.255	0.173

18	I	-1.837	2.326	-0.069
19	I	9.582	-0.312	-0.193
20	C	0.113	3.035	0.124
21	C	7.592	-0.885	0.007
22	Cl	3.242	0.490	2.338
23	Cl	4.573	1.851	-1.888
24	N	-5.315	0.255	-0.173
25	I	1.837	-2.326	0.069
26	C	-1.167	-2.131	-0.154
27	N	-2.759	-3.805	-0.302
28	C	-1.784	-4.695	-0.292
29	C	-0.436	-4.370	-0.198
30	H	-1.020	-1.063	-0.117
31	H	-2.110	-5.723	-0.358
32	H	0.316	-5.145	-0.182
33	Pt	-3.892	-1.160	-0.216
34	N	-2.430	-2.535	-0.230
35	C	-6.594	-0.081	-0.037
36	N	-4.931	1.511	-0.262
37	H	-6.793	-1.137	0.059
38	C	-5.851	2.454	-0.229
39	C	-7.212	2.201	-0.110
40	H	-5.471	3.464	-0.302
41	H	-7.925	3.012	-0.094
42	I	-9.582	0.312	0.193
43	C	-0.113	-3.035	-0.124
44	C	-7.592	0.885	-0.007
45	Cl	-3.242	-0.490	-2.338
46	Cl	-4.573	-1.851	1.888

Dimers: **1.9** [t−$PtCl_2(N_2C_4H_3I)_2$]$_2$ Monomer: t−$PtCl_2(N_2C_4H_3I)_2$

Index	Atom type	*x*	*y*	*z*	Atom type	*x*	*y*	*z*
1	C	0.791	3.345	-3.524	C	2.004	2.093	-0.573
2	N	0.985	4.221	-2.563	N	3.167	2.684	-0.746
3	C	0.649	3.866	-1.328	C	4.263	1.966	-0.533
4	C	-0.044	1.751	-2.102	C	2.944	0.078	-0.001
5	H	1.037	3.623	-4.538	H	1.103	2.667	-0.720
6	H	0.814	4.600	-0.548	H	5.213	2.466	-0.675
7	H	-0.440	0.755	-1.976	H	2.793	-0.957	0.266
8	N	0.297	2.113	-3.342	N	1.845	0.811	-0.211
9	Pt	0.059	0.835	-4.893	Pt	0.000	0.000	0.000
10	C	-0.048	-0.020	-7.712	C	-2.944	-0.078	0.002
11	C	-0.533	-1.722	-6.251	C	-2.004	-2.093	0.573
12	H	0.199	1.021	-7.851	H	-2.793	0.957	-0.266
13	N	-0.735	-2.603	-7.207	N	-3.167	-2.684	0.747
14	H	-0.636	-2.040	-5.224	H	-1.103	-2.667	0.720
15	C	-0.579	-2.198	-8.461	C	-4.263	-1.966	0.533
16	H	-0.744	-2.934	-9.239	H	-5.213	-2.466	0.675
17	N	-0.198	-0.440	-6.451	N	-1.845	-0.811	0.211

18	I	-0.442	2.046	0.890	I	5.914	-0.493	0.195
19	I	0.023	-0.254	-10.733	I	-5.914	0.493	-0.195
20	C	0.113	2.621	-1.041	C	4.197	0.637	-0.146
21	C	-0.225	-0.894	-8.766	C	-4.197	-0.637	0.146
22	Cl	1.390	-0.739	-3.818	Cl	0.686	-1.849	-1.215
23	Cl	-1.216	2.421	-5.982	Cl	-0.686	1.849	1.215
24	N	0.198	0.440	6.451				
25	I	0.442	-2.046	-0.890				
26	C	0.044	-1.751	2.102				
27	C	-0.791	-3.345	3.524				
28	N	-0.985	-4.221	2.563				
29	C	-0.649	-3.866	1.328				
30	H	0.440	-0.755	1.976				
31	H	-1.037	-3.623	4.538				
32	H	-0.814	-4.600	0.548				
33	Pt	-0.059	-0.835	4.893				
34	N	-0.297	-2.113	3.342				
35	C	0.048	0.020	7.712				
36	C	0.533	1.722	6.251				
37	H	-0.199	-1.021	7.851				
38	N	0.735	2.603	7.207				
39	H	0.636	2.040	5.224				
40	C	0.579	2.198	8.461				
41	H	0.744	2.934	9.239				
42	I	-0.023	0.254	10.733				
43	C	-0.113	-2.621	1.041				
44	C	0.225	0.894	8.766				
45	Cl	-1.390	0.739	3.818				
46	Cl	1.216	-2.421	5.982				

Dimers: **1.10** [t−$PtCl_2(N_2C_4H_3I)_2]_2$ Monomer: t−$PtCl_2(N_2C_4H_3I)_2$

Index	Atom type	*x*	*y*	*z*	Atom type	*x*	*y*	*z*
1	C	0.794	3.372	-3.542	C	-0.069	-0.071	-2.937
2	C	0.957	4.234	-2.478	C	-0.632	-2.082	-1.987
3	N	0.646	3.896	-1.231	C	-0.813	-2.609	-3.248
4	C	0.015	1.778	-2.090	N	-0.619	-1.895	-4.352
5	H	1.017	3.653	-4.558	H	0.208	0.962	-2.803
6	H	1.345	5.231	-2.639	H	-0.762	-2.665	-1.089
7	H	-0.355	0.778	-1.934	H	-1.120	-3.639	-3.371
8	N	0.327	2.135	-3.334	Pt	0.000	0.000	0.000
9	Pt	0.071	0.841	-4.865	N	-0.262	-0.804	-1.839
10	C	-0.092	-0.053	-7.662	C	0.069	0.071	2.937
11	C	-0.516	-1.738	-6.163	C	0.632	2.082	1.987
12	H	0.134	0.986	-7.835	H	-0.208	-0.962	2.803
13	C	-0.712	-2.605	-7.217	C	0.813	2.609	3.248
14	H	-0.587	-2.050	-5.134	H	0.762	2.665	1.089
15	N	-0.594	-2.222	-8.484	N	0.619	1.895	4.352
16	H	-0.969	-3.639	-7.033	H	1.120	3.639	3.371
17	N	-0.207	-0.455	-6.396	N	0.262	0.804	1.839

18	I	-0.386	2.083	0.888	I	0.083	0.526	-5.895
19	I	-0.075	-0.305	-10.673	I	-0.083	-0.526	5.895
20	C	0.172	2.681	-1.044	C	-0.248	-0.645	-4.189
21	C	-0.284	-0.963	-8.694	C	0.248	0.645	4.189
22	Cl	1.439	-0.707	-3.805	Cl	-1.212	1.866	-0.643
23	Cl	-1.242	2.410	-5.936	Cl	1.212	-1.866	0.643
24	C	-0.015	-1.778	2.090				
25	C	-0.794	-3.372	3.542				
26	C	-0.957	-4.234	2.478				
27	N	-0.646	-3.896	1.231				
28	H	0.355	-0.778	1.934				
29	H	-1.017	-3.653	4.558				
30	H	-1.345	-5.231	2.639				
31	Pt	-0.071	-0.841	4.865				
32	N	-0.327	-2.135	3.334				
33	C	0.092	0.053	7.662				
34	C	0.516	1.738	6.163				
35	H	-0.134	-0.986	7.835				
36	C	0.712	2.605	7.217				
37	H	0.587	2.050	5.134				
38	N	0.594	2.222	8.484				
39	H	0.969	3.639	7.033				
40	N	0.207	0.455	6.396				
41	I	0.386	-2.083	-0.888				
42	I	0.075	0.305	10.673				
43	C	-0.172	-2.681	1.044				
44	C	0.284	0.963	8.694				
45	Cl	-1.439	0.707	3.805				
46	Cl	1.242	-2.410	5.936				

Dimers: **1.11** $[t-PtCl_2(N_2C_4H_3I)_2]_2$ Monomer: $t-PtCl_2(N_2C_4H_3I)_2$

Index	Atom type	*x*	*y*	*z*	Atom type	*x*	*y*	*z*
1	C	0.813	3.345	-3.391	N	-0.058	-0.126	-2.867
2	C	0.974	4.257	-2.358	C	-0.564	-2.155	-1.884
3	C	0.619	3.866	-1.094	C	-0.747	-2.765	-3.116
4	N	-0.003	1.732	-1.949	C	-0.572	-2.007	-4.244
5	H	1.041	3.575	-4.420	H	-0.660	-2.681	-0.947
6	H	1.362	5.243	-2.565	H	-1.017	-3.809	-3.165
7	H	0.710	4.525	-0.244	H	-0.696	-2.412	-5.236
8	N	0.347	2.126	-3.158	Pt	0.000	0.000	0.000
9	Pt	0.086	0.813	-4.655	N	-0.240	-0.871	-1.793
10	N	0.022	-0.094	-7.380	N	0.058	0.126	2.867
11	C	-0.604	-1.741	-5.885	C	0.564	2.155	1.884
12	C	-0.825	-2.655	-6.904	C	0.747	2.765	3.116
13	H	-0.734	-1.982	-4.842	H	0.660	2.681	0.947
14	C	-0.607	-2.248	-8.194	C	0.572	2.007	4.244
15	H	-1.158	-3.655	-6.669	H	1.017	3.809	3.165
16	H	-0.758	-2.903	-9.039	H	0.696	2.412	5.236
17	N	-0.202	-0.503	-6.146	N	0.240	0.871	1.793

18	I	0.219	-0.187	-10.283	I	0.105	0.595	-5.682
19	C	0.118	2.571	-0.941	I	-0.105	-0.595	5.682
20	I	-0.508	1.852	0.925	C	-0.215	-0.673	-4.048
21	C	-0.171	-0.935	-8.369	C	0.215	0.673	4.048
22	Cl	1.679	-0.628	-3.757	Cl	-1.583	1.611	-0.523
23	Cl	-1.469	2.250	-5.576	Cl	1.583	-1.611	0.523
24	N	0.202	0.503	6.146				
25	I	0.508	-1.852	-0.925				
26	N	0.003	-1.732	1.949				
27	C	-0.813	-3.345	3.391				
28	C	-0.974	-4.257	2.358				
29	C	-0.619	-3.866	1.094				
30	H	-1.041	-3.575	4.420				
31	H	-1.362	-5.243	2.565				
32	H	-0.710	-4.525	0.244				
33	Pt	-0.086	-0.813	4.655				
34	N	-0.347	-2.126	3.158				
35	N	-0.022	0.094	7.380				
36	C	0.604	1.741	5.885				
37	C	0.825	2.655	6.904				
38	H	0.734	1.982	4.842				
39	C	0.607	2.248	8.194				
40	H	1.158	3.655	6.669				
41	H	0.758	2.903	9.039				
42	I	-0.219	0.187	10.283				
43	C	-0.118	-2.571	0.941				
44	C	0.171	0.935	8.369				
45	Cl	-1.679	0.628	3.757				
46	Cl	1.469	-2.250	5.576				

Dimers: **1.12** [t−$PtCl_2(N_3C_3H_2I)_2]_2$ Monomer: t−$PtCl_2(N_3C_3H_2I)_2$

Index	Atom type	x	y	z	Atom type	x	y	z
1	N	1.334	2.774	-2.925	C	0.000	2.846	-0.071
2	N	1.536	3.538	-1.899	N	-1.871	2.109	1.090
3	C	0.880	3.337	-0.754	N	-2.362	3.308	1.091
4	C	-0.282	1.592	-1.754	C	-1.700	4.322	0.535
5	H	1.141	4.008	0.053	H	0.929	2.545	-0.531
6	H	-1.012	0.799	-1.812	H	-2.186	5.286	0.595
7	N	0.435	1.812	-2.852	Pt	0.000	0.000	0.544
8	Pt	0.213	0.610	-4.448	N	-0.702	1.880	0.523
9	C	-0.199	-0.077	-7.253	C	0.000	-2.846	-0.071
10	N	-0.092	-1.896	-5.813	N	1.871	-2.109	1.090
11	H	-0.177	1.000	-7.319	H	-0.929	-2.545	-0.531
12	N	-0.277	-2.711	-6.801	N	2.362	-3.308	1.091
13	C	-0.413	-2.259	-8.048	C	1.700	-4.322	0.535
14	H	-0.555	-3.016	-8.807	H	2.186	-5.286	0.595
15	N	-0.048	-0.596	-6.034	N	0.702	-1.880	0.523
16	I	-0.604	-0.164	-10.259	I	0.577	5.686	-0.972
17	C	-0.062	2.339	-0.614	I	-0.577	-5.686	-0.972

18	I	-1.019	1.873	1.178	C	-0.480	4.138	-0.079
19	C	-0.382	-0.912	-8.335	C	0.480	-4.138	-0.079
20	Cl	2.193	-0.476	-3.904	Cl	0.000	0.000	2.844
21	Cl	-1.758	1.694	-4.985	Cl	0.000	0.000	-1.785
22	N	0.048	0.596	6.034				
23	I	1.019	-1.873	-1.178				
24	I	0.604	0.164	10.259				
25	C	0.282	-1.592	1.754				
26	N	-1.334	-2.774	2.925				
27	N	-1.536	-3.538	1.899				
28	C	-0.880	-3.337	0.754				
29	H	1.012	-0.799	1.812				
30	H	-1.141	-4.008	-0.053				
31	Pt	-0.213	-0.610	4.448				
32	N	-0.435	-1.812	2.852				
33	C	0.199	0.077	7.253				
34	N	0.092	1.896	5.813				
35	H	0.177	-1.000	7.319				
36	N	0.277	2.711	6.801				
37	C	0.413	2.259	8.048				
38	H	0.555	3.016	8.807				
39	C	0.062	-2.339	0.614				
40	C	0.382	0.912	8.335				
41	Cl	-2.193	0.476	3.904				
42	Cl	1.758	-1.694	4.985				

Dimers: **1.13** $[t-PtCl_2(N_2C_3H_3Cl)_2]_2$ Monomer: $t-PtCl_2(N_2C_3H_3Cl)_2$

Index	Atom type	*x*	*y*	*z*	Atom type	*x*	*y*	*z*
1	C	0.688	2.821	-3.014	C	2.573	-1.571	0.517
2	C	0.612	3.184	-1.710	C	3.920	-1.434	0.461
3	C	-0.341	1.208	-1.990	C	2.944	0.451	-0.187
4	H	1.102	3.350	-3.852	H	1.980	-2.422	0.796
5	H	0.950	4.052	-1.176	H	4.730	-2.100	0.687
6	H	-0.839	0.284	-1.763	H	2.817	1.467	-0.512
7	N	0.099	1.589	-3.171	N	1.983	-0.395	0.115
8	Pt	-0.023	0.504	-4.868	Pt	0.000	0.000	0.000
9	Cl	0.385	-1.454	-3.664	Cl	0.433	2.244	0.425
10	Cl	-0.404	2.447	-6.069	Cl	-0.433	-2.244	-0.425
11	Cl	-0.335	2.018	0.572	Cl	5.626	0.564	-0.251
12	C	0.185	-0.133	-7.777	C	-2.944	-0.451	0.187
13	C	-0.575	-1.872	-6.720	C	-2.573	1.571	-0.517
14	H	0.538	0.853	-8.017	H	-2.817	-1.467	0.512
15	C	-0.512	-2.224	-8.027	C	-3.920	1.434	-0.461
16	H	-0.880	-2.450	-5.868	H	-1.980	2.422	-0.796
17	H	-0.756	-3.129	-8.550	H	-4.730	2.100	-0.687
18	N	-0.141	-0.574	-6.582	N	-1.983	0.395	-0.115
19	Cl	0.258	-0.983	-10.323	Cl	-5.626	-0.564	0.251
20	N	-0.047	2.154	-1.086	N	4.129	-0.151	0.012
21	N	-0.028	-1.112	-8.673	N	-4.129	0.151	-0.012

22	C	0.341	-1.208	1.990
23	C	-0.688	-2.821	3.014
24	C	-0.612	-3.184	1.710
25	H	0.839	-0.284	1.763
26	H	-1.102	-3.350	3.852
27	H	-0.950	-4.052	1.176
28	Pt	0.023	-0.504	4.868
29	Cl	-0.385	1.454	3.664
30	Cl	0.404	-2.447	6.069
31	Cl	0.335	-2.018	-0.572
32	N	-0.099	-1.589	3.171
33	C	-0.185	0.133	7.777
34	C	0.575	1.872	6.720
35	H	-0.538	-0.853	8.017
36	C	0.512	2.224	8.027
37	H	0.880	2.450	5.868
38	H	0.756	3.129	8.550
39	N	0.141	0.574	6.582
40	Cl	-0.258	0.983	10.323
41	N	0.047	-2.154	1.086
42	N	0.028	1.112	8.673

Dimers: **1.14** [t−$PtCl_2(N_2C_3H_3Br)_2]_2$ Monomer: t−$PtCl_2(N_2C_3H_3Br)_2$

Index	Atom type	*x*	*y*	*z*	Atom type	*x*	*y*	*z*
1	C	0.672	2.433	-3.153	C	0.121	-0.900	-2.843
2	C	0.566	2.933	-1.895	C	-0.516	-2.559	-1.596
3	C	-0.517	1.024	-2.017	C	-0.487	-3.029	-2.866
4	H	1.159	2.842	-4.018	H	0.419	0.075	-3.184
5	H	0.950	3.828	-1.444	H	-0.759	-3.065	-0.680
6	H	-1.100	0.171	-1.725	H	-0.709	-3.991	-3.286
7	N	0.001	1.236	-3.208	Pt	0.000	0.000	0.000
8	Pt	-0.110	-0.019	-4.778	Cl	-0.511	1.810	-1.367
9	Cl	0.810	-1.766	-3.509	Cl	0.511	-1.810	1.367
10	Cl	-0.945	1.714	-6.051	Br	0.154	-1.982	-5.450
11	Br	-0.502	2.013	0.643	N	-0.138	-1.236	-1.598
12	C	-0.121	-0.889	-7.636	C	-0.121	0.900	2.843
13	C	-0.440	-2.622	-6.369	C	0.516	2.559	1.596
14	H	0.024	0.118	-7.983	H	-0.419	-0.075	3.184
15	C	-0.465	-3.076	-7.646	C	0.487	3.029	2.866
16	H	-0.544	-3.164	-5.448	H	0.759	3.065	0.680
17	H	-0.604	-4.055	-8.063	H	0.709	3.991	3.286
18	N	-0.228	-1.264	-6.379	N	0.138	1.236	1.598
19	Br	-0.183	-1.938	-10.256	Br	-0.154	1.982	5.450
20	N	-0.197	2.032	-1.195	N	-0.080	-1.966	-3.638
21	N	-0.259	-1.965	-8.430	N	0.080	1.966	3.638
22	C	0.517	-1.024	2.017				
23	C	-0.672	-2.433	3.153				
24	C	-0.566	-2.933	1.895				
25	H	1.100	-0.171	1.725				

26	H	-1.159	-2.842	4.018
27	H	-0.950	-3.828	1.444
28	Pt	0.110	0.019	4.778
29	Cl	-0.810	1.766	3.509
30	Cl	0.945	-1.714	6.051
31	Br	0.502	-2.013	-0.643
32	N	-0.001	-1.236	3.208
33	C	0.121	0.889	7.636
34	C	0.440	2.622	6.369
35	H	-0.024	-0.118	7.983
36	C	0.465	3.076	7.646
37	H	0.544	3.164	5.448
38	H	0.604	4.055	8.063
39	N	0.228	1.264	6.379
40	Br	0.183	1.938	10.256
41	N	0.197	-2.032	1.195
42	N	0.259	1.965	8.430

Dimers: **1.15** [t−$PtCl_2(N_2C_3H_3I)_2]_2$ Monomer: t−$PtCl_2(N_2C_3H_3I)_2$

Index	Atom type	*x*	*y*	*z*	Atom type	*x*	*y*	*z*
1	C	1.241	1.804	-2.531	C	-1.444	0.853	2.559
2	C	0.991	2.298	-1.291	C	-1.311	0.798	3.905
3	C	-0.753	1.175	-1.974	C	0.409	-0.203	2.951
4	H	2.119	1.891	-3.144	H	-2.230	1.273	1.960
5	H	1.598	2.888	-0.630	H	-1.929	1.166	4.702
6	H	-1.722	0.710	-1.991	H	1.345	-0.715	2.820
7	N	0.141	1.097	-2.943	N	-0.366	0.228	1.978
8	Pt	-0.005	-0.087	-4.562	Pt	0.000	0.000	0.000
9	Cl	1.795	-1.375	-3.749	Cl	2.286	0.035	0.426
10	Cl	-1.785	1.167	-5.301	Cl	-2.286	-0.035	-0.426
11	I	-1.030	1.833	1.001	I	0.666	-0.308	5.943
12	C	-0.598	-0.915	-7.372	C	-0.409	0.203	-2.951
13	C	0.147	-2.632	-6.278	C	1.444	-0.853	-2.559
14	H	-0.941	0.073	-7.619	H	-1.345	0.715	-2.820
15	C	-0.113	-3.061	-7.536	C	1.311	-0.798	-3.905
16	H	0.538	-3.172	-5.436	H	2.230	-1.273	-1.960
17	H	-0.011	-4.024	-8.000	H	1.929	-1.166	-4.702
18	N	-0.160	-1.295	-6.190	N	0.366	-0.228	-1.978
19	I	-1.160	-1.906	-10.154	I	-0.666	0.308	-5.943
20	N	-0.278	1.899	-0.953	N	-0.132	0.123	4.139
21	N	-0.583	-1.960	-8.219	N	0.132	-0.123	-4.139
22	N	0.160	1.295	6.190				
23	C	0.753	-1.175	1.974				
24	C	-1.241	-1.804	2.531				
25	C	-0.991	-2.298	1.291				
26	H	1.722	-0.710	1.991				
27	H	-2.119	-1.891	3.144				
28	H	-1.598	-2.888	0.630				
29	Pt	0.005	0.087	4.562				

30	Cl	-1.795	1.375	3.749
31	Cl	1.785	-1.167	5.301
32	I	1.030	-1.833	-1.001
33	N	-0.141	-1.097	2.943
34	C	0.598	0.915	7.372
35	C	-0.147	2.632	6.278
36	H	0.941	-0.073	7.619
37	C	0.113	3.061	7.536
38	H	-0.538	3.172	5.436
39	H	0.011	4.024	8.000
40	I	1.160	1.906	10.154
41	N	0.278	-1.899	0.953
42	N	0.583	1.960	8.219

Dimers: **2.1** $[t{-}PdCl_2(NC_5H_4Cl)_2]_2$

Index	Atom type	*x*	*y*	*z*
1	C	1.246	-1.154	2.652
2	C	1.106	-1.474	3.988
3	C	1.319	-0.502	4.948
4	C	1.652	0.770	4.524
5	C	1.759	1.040	3.173
6	H	1.100	-1.880	1.865
7	H	0.840	-2.482	4.266
8	H	1.228	-0.720	6.002
9	H	1.990	2.029	2.808
10	N	1.567	0.084	2.264
11	Pd	1.811	0.451	0.281
12	Cl	0.445	2.329	0.426
13	Cl	3.353	-1.263	0.189
14	C	1.873	-0.354	-2.522
15	C	2.338	1.895	-2.247
16	C	2.055	-0.244	-3.888
17	H	1.609	-1.290	-2.051
18	C	2.542	2.059	-3.602
19	H	2.416	2.716	-1.551
20	C	2.402	0.972	-4.445
21	H	2.811	3.031	-3.988
22	H	2.556	1.061	-5.511
23	N	2.020	0.708	-1.725
24	Cl	1.941	2.039	5.665
25	Cl	1.849	-1.644	-4.882
26	C	-1.652	-0.770	-4.524
27	C	-1.759	-1.040	-3.173
28	C	-1.246	1.154	-2.652
29	C	-1.106	1.474	-3.988
30	C	-1.319	0.502	-4.948
31	H	-1.990	-2.029	-2.808
32	H	-1.100	1.880	-1.865
33	H	-0.840	2.482	-4.266

34	H	-1.228	0.720	-6.002
35	Pd	-1.811	-0.451	-0.281
36	Cl	-0.445	-2.329	-0.426
37	Cl	-3.353	1.263	-0.189
38	N	-1.567	-0.084	-2.264
39	C	-1.873	0.354	2.522
40	C	-2.338	-1.895	2.247
41	C	-2.055	0.244	3.888
42	H	-1.609	1.290	2.051
43	C	-2.542	-2.059	3.602
44	H	-2.416	-2.716	1.551
45	C	-2.402	-0.972	4.445
46	H	-2.811	-3.031	3.988
47	H	-2.556	-1.061	5.511
48	N	-2.020	-0.708	1.725
49	Cl	-1.849	1.644	4.882
50	Cl	-1.941	-2.039	-5.665

Dimers: **2.2** $[t{-}PtCl_2(NC_5H_4Cl)_2]_2$

Index	Atom type	x	y	z
1	C	-1.673	-0.793	-4.522
2	C	-1.788	-1.072	-3.175
3	C	-1.226	1.108	-2.637
4	C	-1.085	1.434	-3.970
5	C	-1.317	0.476	-4.938
6	H	-2.044	-2.057	-2.817
7	H	-1.058	1.824	-1.846
8	H	-0.798	2.439	-4.239
9	H	-1.223	0.699	-5.990
10	Pt	-1.823	-0.498	-0.282
11	Cl	-0.519	-2.422	-0.442
12	Cl	-3.266	1.308	-0.164
13	N	-1.576	-0.127	-2.254
14	C	-1.859	0.311	2.514
15	C	-2.368	-1.931	2.246
16	C	-2.046	0.210	3.878
17	H	-1.572	1.240	2.042
18	C	-2.568	-2.085	3.602
19	H	-2.467	-2.751	1.553
20	C	-2.413	-0.998	4.442
21	H	-2.852	-3.051	3.991
22	H	-2.569	-1.080	5.508
23	Cl	-1.820	1.612	4.865
24	N	-2.029	-0.750	1.716
25	Cl	-1.980	-2.050	-5.671
26	C	1.226	-1.108	2.637
27	C	1.085	-1.434	3.970
28	C	1.317	-0.476	4.938
29	C	1.673	0.793	4.522

30	C	1.788	1.072	3.175
31	H	1.058	-1.824	1.846
32	H	0.798	-2.439	4.239
33	H	1.223	-0.699	5.990
34	H	2.044	2.057	2.817
35	N	1.576	0.127	2.254
36	Pt	1.823	0.498	0.282
37	Cl	0.519	2.422	0.442
38	Cl	3.266	-1.308	0.164
39	C	1.859	-0.311	-2.514
40	C	2.368	1.931	-2.246
41	C	2.046	-0.210	-3.878
42	H	1.572	-1.240	-2.042
43	C	2.568	2.085	-3.602
44	H	2.467	2.751	-1.553
45	C	2.413	0.998	-4.442
46	H	2.852	3.051	-3.991
47	H	2.569	1.080	-5.508
48	N	2.029	0.750	-1.716
49	Cl	1.980	2.050	5.671
50	Cl	1.820	-1.612	-4.865

Dimers: **2.3** [t$-PtCl_2(NC_5H_4F)_2]_2$ Monomer: t$-PtCl_2(NC_5H_4F)_2$

Index	Atom type	x	y	z	Atom type	x	y	z
1	C	-1.586	-0.813	-4.494	C	4.206	0.479	0.272
2	C	-1.729	-1.093	-3.153	C	2.842	0.661	0.335
3	C	-1.260	1.113	-2.628	C	2.508	-1.411	-0.631
4	C	-1.104	1.433	-3.961	C	3.868	-1.628	-0.725
5	C	-1.275	0.458	-4.927	C	4.750	-0.668	-0.263
6	H	-1.955	-2.088	-2.804	H	2.413	1.570	0.727
7	H	-1.134	1.842	-1.843	H	1.790	-2.145	-0.957
8	H	-0.851	2.446	-4.232	H	4.227	-2.550	-1.155
9	H	-1.167	0.663	-5.982	H	5.821	-0.796	-0.311
10	Pt	-1.828	-0.494	-0.263	Pt	0.000	0.000	0.000
11	Cl	-0.534	-2.426	-0.412	Cl	0.256	2.131	-0.877
12	Cl	-3.266	1.316	-0.141	Cl	-0.255	-2.131	0.877
13	N	-1.574	-0.131	-2.239	N	2.007	-0.283	-0.112
14	C	-1.814	0.326	2.525	C	-2.842	-0.661	-0.335
15	C	-2.394	-1.902	2.276	C	-2.508	1.411	0.631
16	C	-1.980	0.221	3.888	C	-4.206	-0.479	-0.272
17	H	-1.510	1.251	2.056	H	-2.413	-1.570	-0.726
18	C	-2.581	-2.045	3.636	C	-3.868	1.628	0.725
19	H	-2.528	-2.721	1.589	H	-1.790	2.145	0.957
20	C	-2.376	-0.962	4.472	C	-4.750	0.668	0.263
21	H	-2.889	-3.001	4.031	H	-4.227	2.550	1.155
22	H	-2.512	-1.025	5.542	H	-5.821	0.796	0.311
23	N	-2.028	-0.734	1.736	N	-2.007	0.283	0.112
24	F	-1.748	1.292	4.644	F	-4.999	-1.442	-0.739
25	F	-1.755	-1.797	-5.373	F	4.999	1.442	0.739

26	C	1.260	-1.113	2.628
27	C	1.104	-1.433	3.961
28	C	1.275	-0.458	4.927
29	C	1.586	0.813	4.494
30	C	1.729	1.093	3.153
31	H	1.134	-1.842	1.843
32	H	0.851	-2.446	4.232
33	H	1.167	-0.663	5.982
34	H	1.955	2.088	2.804
35	N	1.574	0.131	2.239
36	Pt	1.828	0.494	0.263
37	Cl	0.534	2.426	0.412
38	Cl	3.266	-1.316	0.141
39	C	1.814	-0.326	-2.525
40	C	2.394	1.902	-2.276
41	C	1.980	-0.221	-3.888
42	H	1.510	-1.251	-2.056
43	C	2.581	2.045	-3.636
44	H	2.528	2.721	-1.589
45	C	2.376	0.962	-4.472
46	H	2.889	3.001	-4.031
47	H	2.512	1.025	-5.542
48	N	2.028	0.734	-1.736
49	F	1.755	1.797	5.373
50	F	1.748	-1.292	-4.644

Dimers: **2.4** $[t{-}PtCl_2(NC_5H_4Br)_2]_2$

Index	Atom type	*x*	*y*	*z*
1	C	-1.717	-0.784	-4.544
2	C	-1.818	-1.067	-3.196
3	C	-1.210	1.101	-2.655
4	C	-1.078	1.430	-3.987
5	C	-1.341	0.480	-4.957
6	H	-2.086	-2.047	-2.835
7	H	-1.024	1.810	-1.862
8	H	-0.776	2.431	-4.256
9	H	-1.254	0.712	-6.009
10	Pt	-1.819	-0.504	-0.302
11	Cl	-0.509	-2.424	-0.466
12	Cl	-3.265	1.300	-0.186
13	N	-1.577	-0.130	-2.274
14	C	-1.872	0.304	2.495
15	C	-2.353	-1.944	2.224
16	C	-2.066	0.202	3.858
17	H	-1.593	1.233	2.020
18	C	-2.557	-2.101	3.579
19	H	-2.440	-2.766	1.531
20	C	-2.419	-1.011	4.419
21	H	-2.830	-3.071	3.967

22	H	-2.582	-1.099	5.484
23	N	-2.027	-0.760	1.696
24	Br	-1.851	1.736	4.929
25	Br	-2.102	-2.138	-5.795
26	C	1.872	-0.304	-2.495
27	C	2.353	1.944	-2.224
28	C	2.066	-0.202	-3.858
29	H	1.593	-1.233	-2.020
30	C	2.557	2.101	-3.579
31	H	2.440	2.766	-1.531
32	C	2.419	1.011	-4.419
33	H	2.830	3.071	-3.967
34	H	2.582	1.099	-5.484
35	N	2.027	0.760	-1.696
36	C	1.210	-1.101	2.655
37	C	1.078	-1.430	3.987
38	C	1.341	-0.480	4.957
39	C	1.717	0.784	4.544
40	C	1.818	1.067	3.196
41	H	1.024	-1.810	1.862
42	H	0.776	-2.431	4.256
43	H	1.254	-0.712	6.009
44	H	2.086	2.047	2.835
45	N	1.577	0.130	2.274
46	Pt	1.819	0.504	0.302
47	Cl	0.509	2.424	0.466
48	Cl	3.265	-1.300	0.186
49	Br	2.102	2.138	5.795
50	Br	1.851	-1.736	-4.929

Dimers: **2.5** $[t{-}PtCl_2(NC_5H_4I)_2]_2$

Index	Atom type	x	y	z
1	C	-1.801	-0.775	-4.582
2	C	-1.873	-1.062	-3.232
3	C	-1.191	1.083	-2.688
4	C	-1.077	1.418	-4.021
5	C	-1.393	0.483	-4.990
6	H	-2.161	-2.034	-2.864
7	H	-0.970	1.782	-1.894
8	H	-0.752	2.412	-4.290
9	H	-1.323	0.729	-6.040
10	Pt	-1.814	-0.516	-0.337
11	Cl	-0.490	-2.425	-0.506
12	Cl	-3.269	1.281	-0.225
13	N	-1.584	-0.140	-2.309
14	C	-1.895	0.290	2.460
15	C	-2.329	-1.967	2.187
16	C	-2.100	0.193	3.824
17	H	-1.626	1.220	1.980

18	C	-2.541	-2.126	3.541
19	H	-2.396	-2.790	1.494
20	C	-2.433	-1.030	4.379
21	H	-2.797	-3.100	3.928
22	H	-2.606	-1.128	5.441
23	N	-2.022	-0.777	1.660
24	I	-1.922	1.905	5.003
25	I	-2.321	-2.248	-5.968
26	C	1.191	-1.083	2.688
27	C	1.077	-1.418	4.021
28	C	1.393	-0.483	4.990
29	C	1.801	0.775	4.582
30	C	1.873	1.062	3.232
31	H	0.970	-1.782	1.894
32	H	0.752	-2.412	4.290
33	H	1.323	-0.729	6.040
34	H	2.161	2.034	2.864
35	N	1.584	0.140	2.309
36	Pt	1.814	0.516	0.337
37	Cl	0.490	2.425	0.506
38	Cl	3.269	-1.281	0.225
39	C	1.895	-0.290	-2.460
40	C	2.329	1.967	-2.187
41	C	2.100	-0.193	-3.824
42	H	1.626	-1.220	-1.980
43	C	2.541	2.126	-3.541
44	H	2.396	2.790	-1.494
45	C	2.433	1.030	-4.379
46	H	2.797	3.100	-3.928
47	H	2.606	1.128	-5.441
48	N	2.022	0.777	-1.660
49	I	2.321	2.248	5.968
50	I	1.922	-1.905	-5.003

Dimers: **2.6** [t−$PtBr_2(NC_5H_4I)_2]_2$

Index	Atom type	*x*	*y*	*z*
1	C	-1.875	-0.793	-4.591
2	C	-1.966	-1.084	-3.244
3	C	-1.200	1.026	-2.684
4	C	-1.072	1.367	-4.013
5	C	-1.418	0.452	-4.990
6	H	-2.298	-2.046	-2.885
7	H	-0.951	1.712	-1.886
8	H	-0.708	2.350	-4.273
9	H	-1.333	0.700	-6.038
10	Pt	-1.881	-0.567	-0.340
11	Br	-0.423	-2.546	-0.513
12	Br	-3.519	1.253	-0.246
13	N	-1.642	-0.182	-2.312

14	C	-1.922	0.235	2.462
15	C	-2.436	-2.003	2.194
16	C	-2.116	0.146	3.827
17	H	-1.626	1.157	1.983
18	C	-2.640	-2.154	3.550
19	H	-2.542	-2.825	1.504
20	C	-2.486	-1.064	4.387
21	H	-2.928	-3.119	3.939
22	H	-2.652	-1.156	5.451
23	N	-2.090	-0.826	1.662
24	I	-1.863	1.854	5.000
25	I	-2.424	-2.244	-5.988
26	C	1.200	-1.026	2.684
27	C	1.072	-1.367	4.013
28	C	1.418	-0.452	4.990
29	C	1.875	0.793	4.591
30	C	1.966	1.084	3.244
31	H	0.951	-1.712	1.886
32	H	0.708	-2.350	4.273
33	H	1.333	-0.700	6.038
34	H	2.298	2.046	2.885
35	N	1.642	0.182	2.312
36	Pt	1.881	0.567	0.340
37	Br	0.423	2.546	0.513
38	Br	3.519	-1.253	0.246
39	C	1.922	-0.235	-2.462
40	C	2.436	2.003	-2.194
41	C	2.116	-0.146	-3.827
42	H	1.626	-1.157	-1.983
43	C	2.640	2.154	-3.550
44	H	2.542	2.825	-1.504
45	C	2.486	1.064	-4.387
46	H	2.928	3.119	-3.939
47	H	2.652	1.156	-5.451
48	N	2.090	0.826	-1.662
49	I	2.424	2.244	5.988
50	I	1.863	-1.854	-5.000

Dimers: **2.7** $[t{-}PtCl_2(N_2C_4H_3I)_2]_2$

Index	Atom type	*x*	*y*	*z*
1	N	-0.346	2.612	2.202
2	C	-1.482	3.000	2.743
3	C	-2.701	2.981	2.077
4	C	-1.485	2.098	0.249
5	H	-1.402	3.356	3.760
6	H	-3.602	3.328	2.562
7	H	-1.394	1.706	-0.754
8	N	-0.365	2.163	0.966
9	Pt	1.396	1.651	0.136

10	C	4.293	1.508	-0.090
11	N	3.151	0.624	-1.900
12	H	4.182	1.932	0.895
13	C	4.306	0.394	-2.491
14	C	5.539	0.712	-1.935
15	H	4.233	-0.071	-3.464
16	H	6.456	0.504	-2.466
17	N	3.160	1.193	-0.714
18	I	-4.424	2.451	-0.373
19	I	7.273	1.765	0.337
20	C	-2.695	2.516	0.786
21	C	5.525	1.278	-0.684
22	Cl	2.014	0.424	2.010
23	Cl	0.880	2.966	-1.697
24	N	-3.160	-1.193	0.714
25	I	4.424	-2.451	0.373
26	C	1.485	-2.098	-0.249
27	N	0.346	-2.612	-2.202
28	C	1.482	-3.000	-2.743
29	C	2.701	-2.981	-2.077
30	H	1.394	-1.706	0.754
31	H	1.402	-3.356	-3.760
32	H	3.602	-3.328	-2.562
33	Pt	-1.396	-1.651	-0.136
34	N	0.365	-2.163	-0.966
35	C	-4.293	-1.508	0.090
36	N	-3.151	-0.624	1.900
37	H	-4.182	-1.932	-0.895
38	C	-4.306	-0.394	2.491
39	C	-5.539	-0.712	1.935
40	H	-4.233	0.071	3.464
41	H	-6.456	-0.504	2.466
42	I	-7.273	-1.765	-0.337
43	C	2.695	-2.516	-0.786
44	C	-5.525	-1.278	0.684
45	Cl	-2.014	-0.424	-2.010
46	Cl	-0.880	-2.966	1.697

Dimers: **2.8** $[t-PtCl_2(N_2C_3H_3Br)_2]_2$

Index	Atom type	*x*	*y*	*z*
1	C	-1.100	-2.065	-3.916
2	C	-1.227	-2.008	-5.264
3	C	-2.168	-0.226	-4.348
4	H	-0.661	-2.823	-3.295
5	H	-0.915	-2.671	-6.049
6	H	-2.666	0.722	-4.249
7	N	-1.686	-0.951	-3.362
8	Pt	-1.760	-0.468	-1.396
9	Cl	-1.713	1.790	-1.989

10	Cl	-1.838	-2.728	-0.883
11	Br	-2.365	-0.197	-7.163
12	C	-1.520	-0.820	1.563
13	C	-2.099	1.230	1.137
14	H	-1.222	-1.847	1.461
15	C	-2.001	1.135	2.484
16	H	-2.339	2.079	0.526
17	H	-2.149	1.847	3.272
18	N	-1.806	0.007	0.577
19	Br	-1.341	-0.907	4.372
20	N	-1.909	-0.841	-5.515
21	N	-1.638	-0.169	2.729
22	N	1.806	-0.007	-0.577
23	C	2.168	0.226	4.348
24	C	1.100	2.065	3.916
25	C	1.227	2.008	5.264
26	H	2.666	-0.722	4.249
27	H	0.661	2.823	3.295
28	H	0.915	2.671	6.049
29	Pt	1.760	0.468	1.396
30	Cl	1.713	-1.790	1.989
31	Cl	1.838	2.728	0.883
32	Br	2.365	0.197	7.163
33	N	1.686	0.951	3.362
34	C	1.520	0.820	-1.563
35	C	2.099	-1.230	-1.137
36	H	1.222	1.847	-1.461
37	C	2.001	-1.135	-2.484
38	H	2.339	-2.079	-0.526
39	H	2.149	-1.847	-3.272
40	Br	1.341	0.907	-4.372
41	N	1.909	0.841	5.515
42	N	1.638	0.169	-2.729

© 2019 by the authors. Submitted to *Materials* for possible open access publication under the terms and conditions of the Creative Commons Attribution (CC BY) license (http://creativecommons.org/licenses/by/4.0/).
